# Rose Bengal-Incorporated Supramolecular Gels as a Topical Platform for Localized Antimicrobial Photodynamic Therapy

**DOI:** 10.3390/ijms262311455

**Published:** 2025-11-26

**Authors:** Kavya Anguluri, Saman Bagherpour, Ana C. Calpena, Lyda Halbaut, Alba Espargaró, Raimon Sabate, Lluïsa Pérez-García

**Affiliations:** 1 Departament de Farmacologia, Toxicologia i Química Terapèutica, Facultat de Farmàcia i Ciències de l’Alimentació, Universitat de Barcelona, Avda. Joan XXIII 27-31, 08028 Barcelona, Spain; nvangula89@alumnes.ub.edu (K.A.); saman.bagherpour@ub.edu (S.B.); 2 Institut de Nanociència i Nanotecnologia IN2UB, Universitat de Barcelona, Avda. Joan XXIII 645, 08028 Barcelona, Spain; anacalpena@ub.edu; 3 Departament de Farmàcia, Tecnologia Farmacèutica, i Fisicoquímica, Facultat de Farmàcia i Ciències de l’Alimentació, Universitat de Barcelona, Avda. Joan XXIII 27-31, 08028 Barcelona, Spain; halbaut@ub.edu (L.H.); aespargaro@ub.edu (A.E.); rsabate@ub.edu (R.S.); 4 Institut de Biomedicina (IBUB), Universitat de Barcelona, Avda. Joan 643, 08028 Barcelona, Spain

**Keywords:** supramolecular gels, rose bengal, singlet oxygen generation, photoactivated antimicrobial response, photodynamic therapy

## Abstract

Efficient and localized singlet oxygen (SO) generation is essential for improving antimicrobial photodynamic therapy (aPDT). In this study, a bis-imidazolium-based amphiphilic gelator is used, which self-assembles into a supramolecular gel in a water–ethanol medium and incorporates Rose Bengal (**RB**) as a photosensitizer. The gel network provides a confined environment that promotes SO formation under light irradiation. **RB**@Gel was characterized with respect to its morphology, degradation behavior, and swelling properties. Biopharmaceutical assessment included in vitro release, ex vivo permeation studies and Hen’s Egg Test–Chorioallantoic Membrane (HET-CAM) assay. Rheological measurements confirmed a viscoelastic profile, indicating structural stability and suitability for localized therapeutic applications. SO production within the gel was quantified using tetrasodium 9,10-anthracenediyl-bis(methylene)dimalonate (NaABMA), showing higher efficiency than that of **RB** in solution. The **RB**@Gel exhibited significant aPDT against *E. coli* in a direct-surface contact assay. Overall, the **RB**@Gel provides a stable, suitable platform capable of efficient SO generation and potent antibacterial activity, highlighting its promise for localized aPDT applications.

## 1. Introduction

Xanthene derivatives represent a class of organic dyes characterized by a tricyclic aromatic structure containing an oxygen atom in the central ring. These dyes exhibit strong absorption in the visible green region and are well known for their photosensitizing capability. Owing to these properties, xanthene-based compounds have been widely employed in photodynamic therapy (PDT) [1,2,3]. Among the various xanthene-based dyes, Rose Bengal (**RB**) stands out as a well-known, water-soluble photosensitizer (PS) that is both economical and clinically relevant. It is recognized for its strong ability to generate reactive oxygen species (ROS) and for possessing a high singlet-oxygen (SO) quantum yield [4,5,6]. In PDT, a PS, light of a suitable wavelength, and molecular oxygen interact to produce ROS such as SO and free radicals which cause oxidative damage to microbial cell membranes, proteins, and nucleic acids, ultimately leading to cell death. When PDT is applied to microorganisms, it is referred to as antimicrobial PDT (aPDT), which is a promising strategy for the inactivation of pathogens [7,8]. Unlike conventional antibiotics, this oxidative mechanism targets multiple cellular components, thereby minimizing the potential for resistance development [6,7,8]. Recent advances in molecular photosensitizer design have yielded multifunctional systems capable of NIR-II emission, aggregation-induced photosensitivity, and controllable photodegradation for improved safety and efficacy. For instance, a self-degrading A–D–A-type photosensitizer exhibiting simultaneous type I/II photosensitivity and photothermal effects, effective for both antibacterial and anticancer phototherapy has been reported [9]. Likewise, aggregation-induced type I/II photosensitizers with photodegradable backbones that enhance therapeutic outcomes while reducing phototoxicity [10]. These studies highlight current strategies in designing degradable and multifunctional PSs.

The rapid rise in antimicrobial resistance among disease-causing bacteria represents a major global health concern, prompting the search for alternative therapeutic strategies to conventional antibiotics that can effectively eradicate infections without promoting resistance [11,12,13]. PDT has emerged as a promising non-invasive approach for antimicrobial treatment [14,15,16]. A key advantage of this approach lies in its broad-spectrum activity, targeting both Gram-positive and Gram-negative bacteria [17,18]. *Escherichia coli* (*E. coli*) has been identified as a causative agent in multiple skin and soft tissue infections (SSTIs), particularly in wound infections [19,20,21,22]. Although *E. coli* is primarily an intestinal commensal, certain extraintestinal pathogenic strains (ExPEC) have been described in association with infections of compromised skin or wound sites, sometimes resulting in cellulitis, abscess formation, and, in rare cases, necrotizing infections. These infections were observed in immunocompromised and hospitalized patients, where impaired immune defenses and frequent antimicrobial exposure promote colonization and resistance [23,24,25]. While *E. coli* is an uncommon cause of primary cutaneous infection, its occasional implication in lesions such as ecthyma gangrenosum [26,27], underscore its potential to cause serious cutaneous infections even outside the gastrointestinal tract. The growing prevalence of multidrug-resistant *E. coli* isolates in wound and soft-tissue infections underscores the urgent need for localized, non-antibiotic therapeutic approaches capable of achieving effective bacterial eradication while minimizing systemic side effects [28,29,30]. The growing rise in bacterial resistance has significantly reduced the effectiveness of many existing antibiotics, underscoring the urgent need for new therapeutic strategies. In this context, gels have attracted attention due to their favorable characteristics, such as thixotropic properties, prolonged retention on the application site, and ease of spreading, making them particularly suitable for topical therapeutic use [17,18,31]. For example, a fluconazole topical gel formulation demonstrated good spreadability, rheological behavior and favorable topical performance, highlighting the suitability of gel-based systems for localized topical therapy [31].

Nanoparticle-based delivery systems, including polymeric nanoparticles, liposomes, and coordination polymer nanostructures, have been widely investigated to improve PS solubility, stability, and overall photodynamic performance. Nevertheless, their use in topical PDT remains limited due to low incorporation efficiency and poor retention of PS at the application site. Furthermore, many carrier materials reported in the literature exhibit suboptimal degradability and biocompatibility, restricting their clinical translation [32,33,34]. For instance, Hypericin-loaded solid lipid nanoparticles improved photostability but showed reduced phototoxicity due to quenching within the compact lipid matrix, demonstrating how nanoparticle carriers can sometimes compromise PS performance despite improved solubility [32]. In contrast, supramolecular hydrogels provide a biocompatible and tunable platform for incorporating therapeutic agents through reversible, non-covalent interactions such as hydrogen bonding, π–π stacking, and hydrophobic effects. Their assembly from low-molecular-weight gelator results in physically crosslinked networks with facile degradability, high flexibility, and dynamic responsiveness [35]. Compared with conventional polymeric gels, supramolecular hydrogels offer distinct advantages, including thermo-reversibility and structural adaptability governed by weak intermolecular forces rather than covalent bonds. Despite these benefits, the application of supramolecular hydrogels as matrices for PDT organization in aPDT is relatively underexplored [32]. A fluorenylmethoxycarbonyl-diphenylalanine (Fmoc-FF)/C_60_-PTC fullerene hydrogel achieved complete *S. aureus* inactivation and nearly 100% wound closure within seven days in an infected murine skin model under white-light irradiation [36], while fluorenylmethoxycarbonyl-L-phenylalanine/berberine (Fmoc-F/BBR) hydrogel exhibiting aggregation-induced emission showed 36.9% antibacterial activity against *E. coli* and 100% against *S. aureus* in vitro, along with approximately 95% wound closure after 10 days in vivo [37].

**Figure 1:**
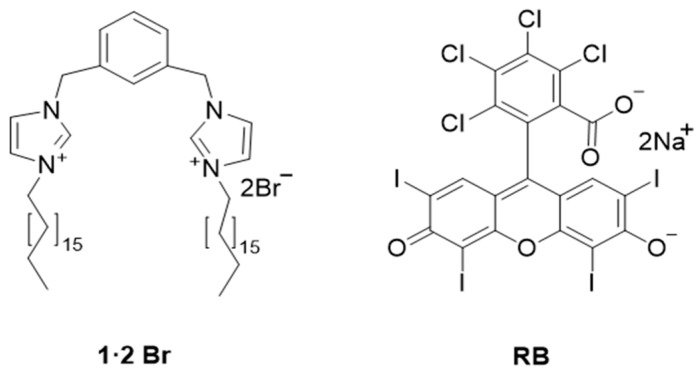
Molecular structures of the amphiphile **1·2 Br** and the photosensitizer **RB** used in this study.

Our research group had recently reported that bis-imidazolium-based supramolecular gels incorporating porphyrin PS’s significantly enhance SO generation and exhibit potent aPDT against *S. aureus* and *E. coli* [34]. The molecular structures of gemini imidazolium-based amphiphile **1·2 Br** (1,3-bis[(3-octadecyl-1-imidazolio)methyl]benzene dibromide) and photosensitizer **RB** used in this study are shown in Figure 1. In this study, we prepared and characterized an **RB**-incorporated gel (**RB**@Gel), which was evaluated for its physicochemical stability and rheological properties to assess its suitability as a topical delivery system. The release kinetics of **RB** from the gel matrix were analyzed under conditions with and without a membrane barrier to better understand its release behavior. Furthermore, ex vivo skin permeation studies using dorsal pig skin were performed to simulate topical delivery conditions. Accordingly, aPDT experiments were conducted against *E. coli* to evaluate the aPDT efficacy of the prepared PS-loaded gel. Compared with the previously reported supramolecular hydrogels, our hydrogel demonstrates a simpler and more cost-effective preparation method, offering better scalability. The developed gels demonstrated high encapsulation efficiency, mechanical integrity, and prolonged PS retention which may minimize diffusion to healthy tissue and reducing the need for repeated application. Owing to their intrinsic biocompatibility and structural tunability, such supramolecular hydrogels represent a promising platform for localized aPDT.

## 2. Results and Discussion

### 2.1. Self-Assembly of Gels

**Figure 2:**
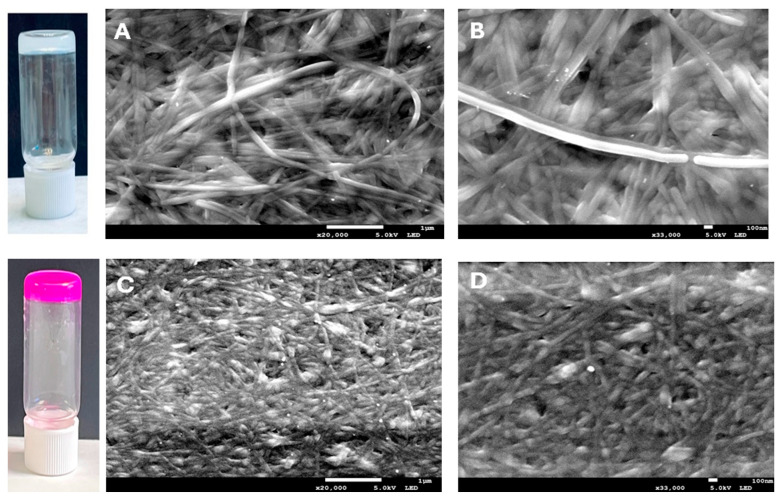
Photographs of the **1·2 Br**@Gel and **RB**@Gel in inverted vials demonstrating their stable formation. Concentration: **RB** = 1 mM, **1·2 Br** = 12 mM (water: ethanol 1:1). SEM micrographs of xerogels prepared from **1·2 Br** gels (12 mM) are shown for samples without the photosensitizer (Images (**A**,**B**)) and with **RB** (0.3 mM) (Images (**C**,**D**)). Scale bars correspond to 1 μm (**left column**) and 100 nm (**right column**).

The gemini imidazolium-based amphiphile **1·2 Br** acted as an efficient molecular gelator, forming quickly supramolecular gels via self-assembly in water–ethanol mixtures at room temperature. Due to its solubility in ethanol, the addition of water as an anti-solvent initiates the self-assembly process and enables fiber network formation [38]. Gelation occurred upon mixing water with an ethanolic solution of **1·2 Br**, resulting in a final amphiphile concentration of 12 mM and a 1:1 (*v*/*v*) water-to-ethanol ratio, as evidenced by the stable gel formation shown in Figure 2. These conditions were selected based on our previous findings, where a 12 mM amphiphile concentration provided the highest PS loading efficiency, and a 1:1 solvent ratio yielded well-formed hybrid gels [39].

### 2.2. Gels Characterization

#### 2.2.1. Morphological Analysis

Scanning Electron Microscopy (SEM) images (Figure 2) show that the supramolecular gel (**1·2 Br**@Gel) forms a dense, entangled fibrillar network, with fibers ranging from 50 to 220 nm in width. This structure reflects the typical self-assembly of gelator molecules into fibrous aggregates that interconnect to give the gel its solid-like properties (Figure 2A,B). When **RB** is incorporated into the gel, the overall fibrillar morphology is maintained, but the network looks slightly more compact and uniform (Figure 2C,D). This suggests that **RB** molecules interact with the gel matrix likely embedding within the fibers, without disturbing the main assembly process. Instead, their presence appears to promote a tighter packing of the network, making it more homogeneous.

#### 2.2.2. Determination of pH and Density

The pH values of both gels fall within the physiological acidic range of healthy human skin (pH 4–6), ensuring their suitability for topical administration. The **1·2 Br**@Gel exhibited a pH of 5.9 ± 0.2, whereas the **RB**@Gel showed a lower value of 5.2 ± 0.1. Statistical analysis confirmed that the difference between the two gels was significant (*p* = 0.0056) (Table S1), with the **RB**@Gel demonstrating a more pronounced acidity, likely attributable to the presence of **RB**. Such acidic microenvironments are advantageous, particularly when considering application to chronic or infected wounds, which frequently display elevated alkalinity, suppresses bacterial proliferation [40,41,42]. Density measurements further differentiated the structural characteristics of the hydrogels. The **1·2 Br**@Gel presented a density of 0.954 ± 0.028 g/mL, whereas the **RB**@Gel reached 1.159 ± 0.030 g/mL. This difference was statistically significant (*p* = 0.0077) (Table S1). The noticeably higher density of the **RB**@Gel suggests a more tightly packed molecular arrangement, consistent with enhanced intermolecular interactions between **1·2 Br** and **RB**. This interpretation aligns with the morphological features observed by SEM, which revealed a more compact fibrillar network in the **RB**@Gel.

#### 2.2.3. Swelling and Degradation Tests

**Figure 3:**
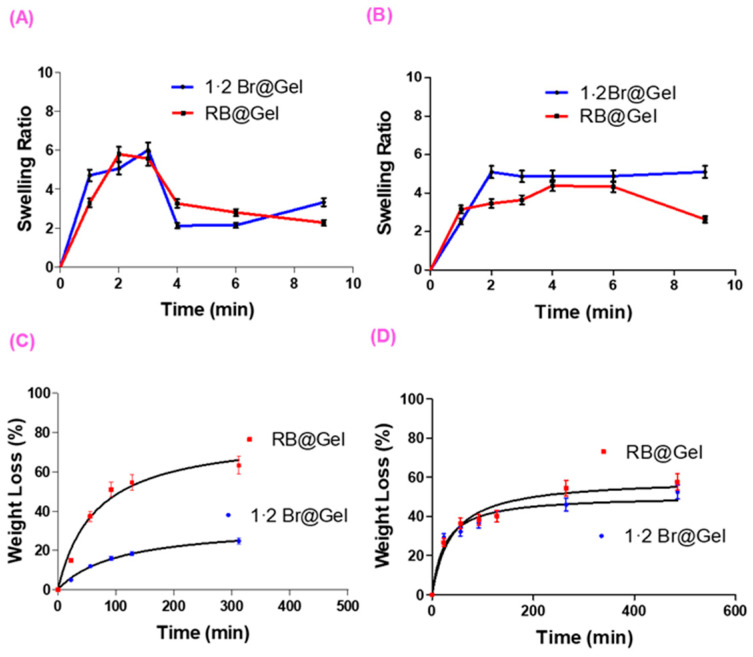
Swelling and Degradation behavior of **1·2 Br**@Gel and **RB**@Gel at different pH conditions. Swelling ratio as a function of time at pH 5.5 (**A**) and 7.4 (**B**). Corresponding weight loss profiles representing gel degradation at pH 5.5 (**C**) and 7.4 (**D**). Data were expressed as mean ± SD from three independent measurements.

At pH 5.5 (Figure 3A), both **1·2 Br**@Gel and **RB**@Gel exhibited rapid initial swelling (maximum ratio 6), followed by a collapse to lower values (2–3) within 6–8 min. The swelling ratio (*SR*) was determined gravimetrically as the ratio of the swollen gel weight (Wₜ) to its initial dry weight (W_0_). This collapse reflects destabilization of the supramolecular network under acidic conditions, and the similar profiles despite statistically significant differences (Table S2) indicate that pH effects outweigh any **RB**–gelator interactions. At pH 7.4 (Figure 3B), the **1·2 Br**@Gel swelled rapidly to a stable ratio of approximately 5, whereas **RB**@Gel reached a lower maximum (4) and subsequently underwent gradual deswelling. As observed in Table S3, statistically significant differences are present at 2, 3, and 9 min, with the **RB**@Gel exhibiting a lower swelling capacity. This reduced swelling capacity is consistent with **RB**–gelator interactions that compact the fibrillar network and limit water uptake. Similar pH-dependent swelling–deswelling behavior has been observed in peptide- and low-molecular-weight supramolecular gels, where acidic environments disrupt hydrogen-bonded or ionic assemblies [43,44]. Moreover, the decreased swelling in **RB**@Gel aligns with reports that electrostatic interactions between anionic dyes and cationic gel matrices can promote tighter molecular packing and reduced solvent uptake [35]. Such interactions may enhance network stability and promote localized retention of the photosensitizer within the gel matrix and at the superficial layers of the skin under physiological pH conditions, where electrostatic and hydrophilic–hydrophobic balance restricts deeper diffusion. This behavior is advantageous for topical aPDT, as it maintains a high local photosensitizer concentration at the target surface without systemic exposure [45].

The modeling of the degradation kinetics of the gels at the different pH values studied will allow us to accurately determine the percentage of degradation as a function of time. Specifically, at pH 5.5, the model that provided the best fit among the many tested was a hyperbolic model, and the same model type was found to be the most appropriate at pH 7.4 (Tables S4 and S5). In addition, a statistical analysis of the model parameters, **Bmax** and **Kd**, was carried out using a Student’s *t* test to assess the influence of **RB** on these parameters. The degradation of supramolecular gels at pH 5.5 was assessed by monitoring weight loss over time (Figure 3C). While the **1·2 Br**@Gel degraded slowly (25% loss after 300 min), **RB**@Gel showed a more dynamic profile with 60% loss in the same period. This accelerated breakdown arises from electrostatic interactions between **RB** and the cationic imidazolium groups, which facilitate partial matrix disassembly. Consistent with this observation, the **Bmax** values showed statistically significant differences, with higher values obtained for the **RB**@Gel. At pH 7.4, degradation was similar for both gels (Figure 3D), and no statistically significant differences were observed in either of the model parameters.

These findings are consistent with the pH-dependent swelling behavior, confirming that acidic conditions weaken the supramolecular network through disruption of electrostatic **RB**–gelator interactions. At physiological pH, the gels exhibited slow and partial degradation, indicating that the network remains sufficiently stable for topical application while gradually disassembling over time. This moderate degradation supports localized retention of **RB** within the gel matrix and superficial skin layers, promoting targeted photodynamic action at the infected site while minimizing diffusion and systemic exposure [35,46]. This behavior demonstrates that the gel system maintains structural persistence at the application site during the treatment phase—regardless of whether it is applied to healthy or inflamed skin—while the gradual degradation observed under physiological conditions ensures localized **RB** retention without prolonged accumulation or systemic diffusion. Such balance between stability and degradability is advantageous for localized PDT applications [45].

#### 2.2.4. Extensibility and Rheological Tests

**Figure 4:**
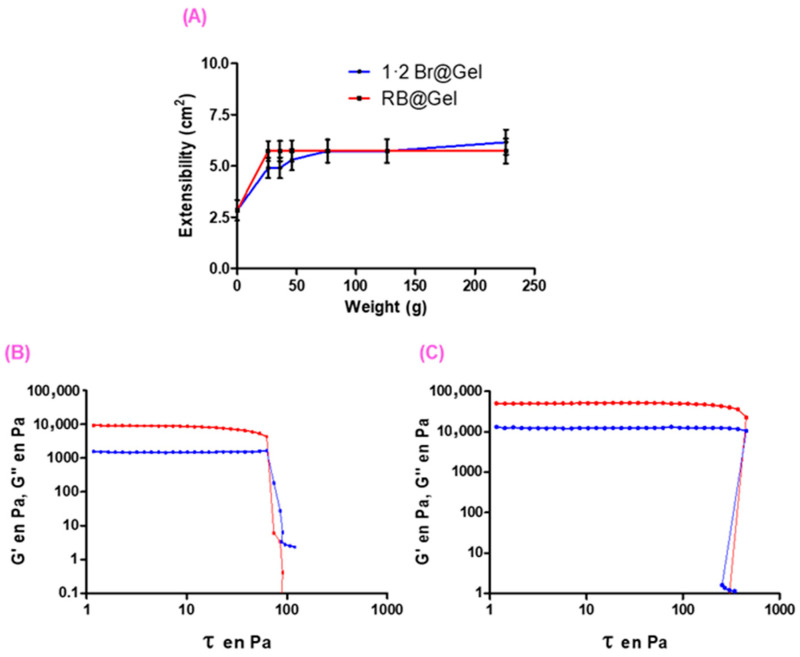
Extensibility profiles (**A**) of the **1·2 Br**@Gel and **RB**@Gel. The plot shows the change in spread area (cm^2^) as a function of the applied weight (g). Shear Stress profiles displaying the storage (G′, red curve) and loss (G″, blue curve) moduli from gels of 12 mM **1·2 Br**@Gel (**B**) and with 1 mM **RB** concentration (**C**).

The extensibility of both the **1·2 Br**@Gel and **RB**@Gel was evaluated under varying applied weights (Figure 4A). No statistically significant differences were observed (Table S6A). When compared with commercial formulations, our gels showed no statistically significant differences in extensibility relative to Canesten and SolvEasy Tinea (Table S6B). However, significant differences were found when compared with Voltaren and Ureadin Podos, indicating that our formulations are less soft and deformable than these commercial products. The lower extensibility of both the **1·2 Br**@Gel and **RB**@Gel reflects greater consistency and cohesive strength which are desirable characteristics for maintaining the gel integrity during topical application. In the context of aPDT, this mechanical consistency ensures that the PS remains localized at the treatment site, with minimizing flow during irradiation and enabling uniform light exposure and SO generation at the infected surface [47,48].

The mechanical characteristics of the gels were further examined through rheological analysis as a function of **RB** loading. Both the **1·2 Br**@Gel and **RB**@Gel exhibited $G^{\prime }>G^{\prime \prime }$ across the entire frequency range, confirming a predominantly elastic nature of these gels [35]. Notably, **RB**@Gel displayed higher $G^{\prime }$ values, indicative of a stiffer network arising from electrostatic interactions between **RB** and the gelator, which strengthen the fibrous matrix (Figure 4B,C). The rheological data derived from the stress-sweep tests are summarized in Table 1. Both gel systems, with and without **RB** displayed broad linear-viscoelastic behavior and high moduli at low shear stress. **1·2 Br**@Gel exhibited a critical stress value (CSV) of 63 Pa, whereas **RB**@Gel showed a significantly higher CSV of 423 Pa. This behavior can be attributed to electrostatic interactions between **RB** and the positively charged surface of the gel fibers. The strong viscoelastic behavior of **RB**@Gel further indicates stable dye entrapment within the supramolecular network, consistent with the negligible release observed in release studies (see Section 2.2.5). This mechanical resilience a key advantage for topical aPDT by ensuring that **RB** remains firmly localized and concentrated at the treatment site during light exposure, thereby maintaining effective therapeutic contact similar to that reported for porphyrin-loaded hydrogels designed for topical PDT [49].

#### 2.2.5. Release Studies of **RB** from **RB**@Gel

The release of **RB** from the gel was evaluated using both the dialysis membrane method and the direct-surface contact method. The dialysis membrane method showed no release of **RB** from the gel, whereas the direct-surface contact method resulted in a cumulative release of approximately 3 µg (2%) of **RB** after 43 h (Figure S1), which is considered negligible diffusion from the gel matrix. The best-fitting model describing the release kinetics of **RB** from the gel using the latter method was a double-hyperbolic model; the corresponding parameters are shown in Table S7. Such restricted diffusion is desirable for topical aPDT as effective treatment requires that the PS remain localized at the wound site to enable site-specific SO generation during irradiation. Similar behavior has been reported for PS-loaded hydrogels designed to fix the active compound at wound sites and enable localized photodynamic action [48,50]. This strong retention suggests that **RB**@Gel can act as a stable and site-specific platform for controlled photodynamic therapy.

#### 2.2.6. Hen’s Egg Test–Chorioallantoic Membrane (HET-CAM) Irritancy Test

**Figure 5:**
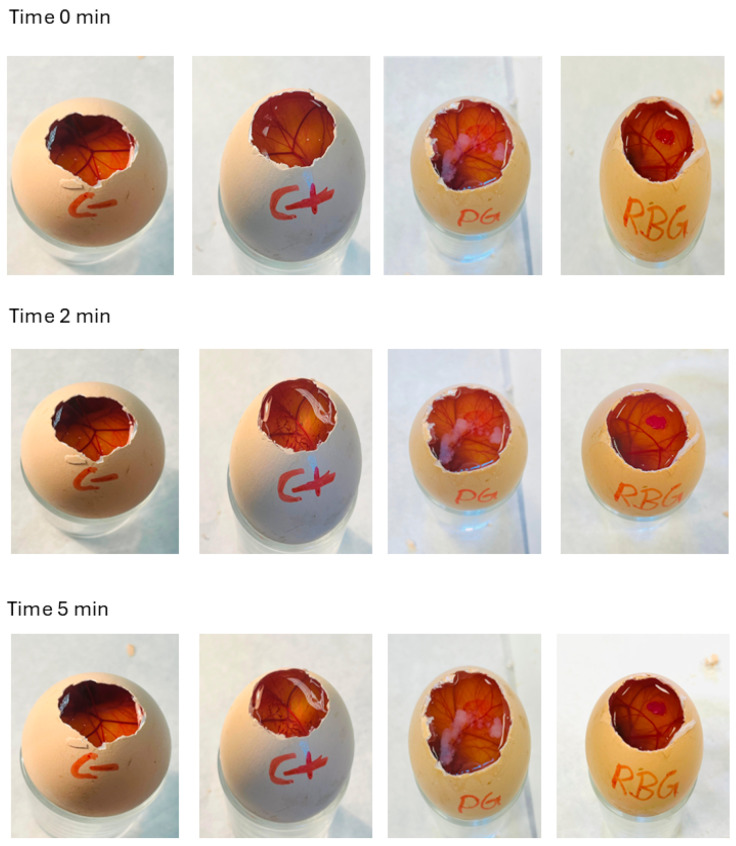
HET-CAM assay results showing the irritation potential of the tested gels on the CAM of fertilized chicken eggs. Images were captured at 0, 2, and 5 min following application of the samples. C−: negative control (0.9% NaCl solution); C+: positive control (0.1 M NaOH); In this figure, PG: **1·2 Br**@Gel; RBG: **RB**-incorporated gel (**RB**@Gel).

HET-CAM assay is a widely accepted in vitro method for assessing the irritation potential of formulations intended for ocular or topical application, was employed to evaluate the irritancy potential of the gels (Figure 5). The irritation potential was quantified using the irritation score (IS), calculated based on the time of onset and severity of hemorrhage, vessel lysis, and coagulation on the Chorioallantoic membrane. **1·2 Br**@Gel caused vascular redness and slight coagulation immediately after application (0 min), which persisted up to 5 min (*IS* = 1), indicating a slightly irritant response. In contrast, the **RB**@Gel showed no observable irritant effects throughout the 0–5 min observation period (*IS* = 0), indicating excellent tolerability. As expected, the negative control (0.9% NaCl) produced no irritation, while the positive control (0.1 M NaOH) induced irritation beginning at 2 min and intensifying by 5 min. The absence of irritation in **RB**@Gel confirms that the incorporation of **RB** into the gel matrix did not trigger any adverse vascular response, and RB@Gel can be classified as non-irritant under the tested conditions. This observation agrees with previous studies where methylene blue-based hydrogels developed for aPDT exhibited a *IS* of zero, further supporting the safety of PS-loaded topical formulations [51,52]. The observed biocompatibility is advantageous for aPDT, ensuring that photosensitizer activation and SO generation remain confined to the targeted infection site without inducing off-target tissue irritation, thereby maintaining the efficacy of aPDT after topical application.

### 2.3. Ex Vivo Skin Permeation Studies of **RB**@Gel

**RB**@Gel was applied to dorsal pig skin mounted in Franz-type diffusion cells to assess its permeation and retention profile. The receptor compartment contained water and was maintained at 32 ± 1 °C under continuous stirring to mimic physiological skin conditions. Samples from the receptor medium were collected at 24 h and analyzed spectrophotometrically to quantify the amount of **RB** permeated through the skin. The permeation of **RB** through dorsal pig skin was zero. In contrast, 0.88% of **RB** was retained in the skin. Because the stratum corneum and viable layers were not separated, this value represents the total skin retention, with most of the remaining dose most likely present on top of the skin. Given **RB**’s hydrophilic xanthene structure and anionic character at physiological pH, its diffusion across the lipophilic stratum corneum barrier is inherently limited, restricting its penetration into deeper skin layers [6,53]. This retention indicates that the supramolecular gel efficiently restricts **RB** penetration beyond the outer skin barrier, promoting surface-level localization. Such distribution is advantageous for topical aPDT, ensuring a high local photosensitizer concentration at the infection site without systemic exposure.

### 2.4. Optical Properties and SO Production of **RB** in Solution and in **RB**@Gel

**Figure 6:**
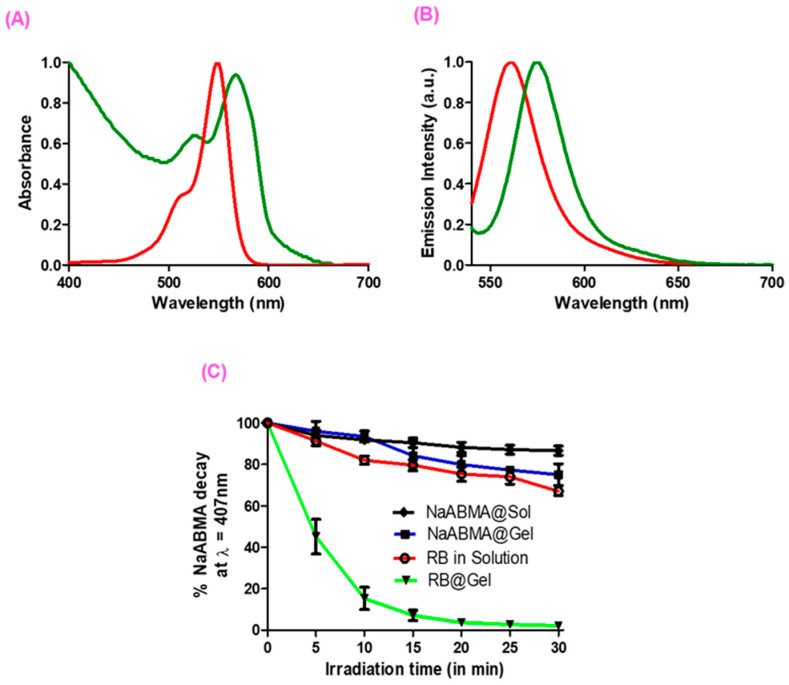
Normalized absorption spectra (**A**) and emission spectra (**B**) of **RB** in solution (red line) and in gel (green line). Experimental conditions: **RB** = 2 µM, **1·2 Br** = 12 mM. (**C**) Normalized plot of percentage decay of NaABMA emission upon irradiation of NaABMA and **RB** both in solution and in gel. Experimental conditions: **RB** = 2 μM, NaABMA = 5 μM, PBS = 2 mM at pH = 7.4.

Figure 6 shows the images of **1·2 Br**@Gel and **RB**@Gel. Figure 6A,B show the absorption and fluorescence spectra of **RB** in solution (1:1 water: ethanol) (red line) and in the gel (green line). The absorption spectrum of **RB** in solution exhibits characteristic features of xanthene dyes, with an intense absorption band centered at λ = 548 nm in the visible region [54]. These spectral characteristics remain largely unchanged when **RB** is incorporated into the gel matrix. The absorption spectrum of **RB**@Gel displays a slight elevation in the baseline, attributed to light scattering caused by the fibrous structure of the matrix, along with a modest red shift of +18 nm, resulting in an absorption maximum at λ = 566 nm. The fluorescence spectacle exhibits a comparable red-shift behavior. **RB** in solution shows a single, intense emission band at λ = 562 nm, which undergoes a slight red shift upon gel incorporation. In **RB**@Gel, the emission maximum appears at λ = 574 nm, corresponding to a bathochromic shift of +12 nm, consistent with the shift observed in the absorption spectra. The observed bathochromic shift in RB within the gel matrix can be attributed to the polar and charged microenvironment created by the imidazolium network, which stabilizes the excited state of the dye and slightly reduces the energy gap between the ground and excited states, consistent with field-effect–induced shifts reported for xanthene dyes [55]. Moreover, the confined structure of the gel likely limits dye–dye interactions, thereby reducing aggregation and preserving the monomeric, photoactive form of **RB**, in agreement with previous findings on encapsulated **RB** systems [56].

The SO generation capacity of both **RB** in solution and **RB**@Gel were assessed using tetrasodium 9,10-anthracenediyl-bis(methylene)dimalonate (NaABMA) as a detection probe under irradiation using λ = 532 nm laser for 30 min. Oxidation of NaABMA by SO leads to a measurable decrease in its characteristic emission band and the emission spectra was recorded every 5 min intervals [57]. Figure 6C presents the comparative results of these experiments. Control samples revealed a 26% decrease in NaABMA in solution and a 28% decrease in the **1·2 Br**@Gel after 30 min of irradiation. The latter effect may be attributed to minor interactions between the gel matrix and incident light, as the fibrillar network could promote low-level photochemical processes even in the absence of a photosensitizer. In contrast, incorporation of **RB**@Gel resulted in a substantial decrease in NaABMA emission, with up to 98% degradation, compared to only 26% when **RB** was used in solution. These findings highlight the markedly enhanced SO generation efficiency of **RB** upon encapsulation within the gel. Figure S2 shows the emission spectra of NaABMA recorded after the addition of **RB** in solution and **RB**@Gel, followed by 30 min of irradiation.

### 2.5. Antibacterial Studies

**Figure 7:**
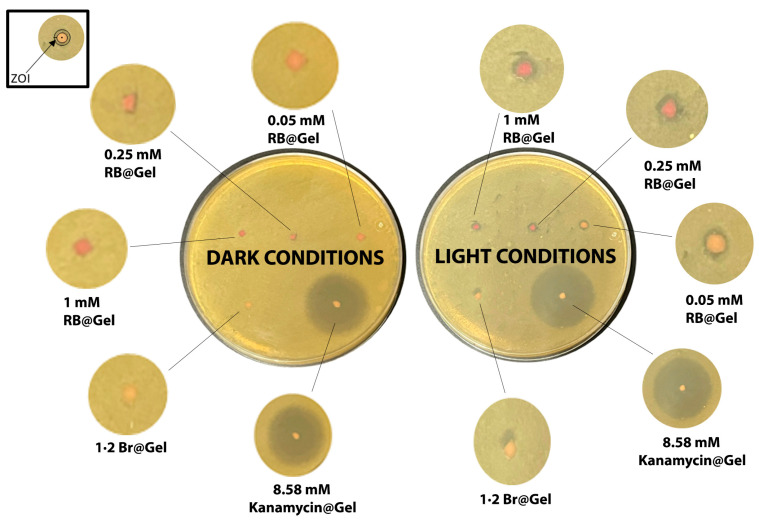
Representative agar plate images illustrating the aPDT activity of **RB**@Gel under dark and light conditions. The concentration of the **1·2 Br** is 12 mM in all gels. The Zone of Inhibition (ZOI) was measured as the width of the clear halo around the gel, excluding the diameter of the gel itself.

The antibacterial photodynamic therapy (aPDT) efficacy of **RB**@Gel was evaluated against *E. coli* using the direct-surface contact method (Figure 7). Bacterial suspensions were evenly spread on nutrient agar plates, and gel samples containing different **RB** concentrations (1 mM, 0.25 mM, and 0.05 mM) were placed directly on the agar surface. The samples were irradiated with a 532 nm laser for 30 min, and the aPDT efficacy was determined by measuring the zone of inhibition (ZOI). Upon irradiation, all **RB**@Gel samples produced a ZOI of approximately 1.4 mm, whereas the **1·2 Br**@Gel control showed no inhibition.

The ZOI did not increase proportionally with higher **RB** concentrations, suggesting that the antibacterial effect is influenced by both the diffusion of **RB** and the local concentration of SO. While the overall antibacterial response depends on the amount of SO produced—which increases with the local concentration of **RB**, the spatial extent of the inhibition zone is primarily limited by the restricted diffusion of both **RB** and SO within the gel matrix. The restricted mobility of **RB** from the supramolecular gel network, as confirmed by both the dialysis membrane and direct-surface contact release studies, limits its diffusion into the agar medium. Moreover, the short lifetime (<200 ns) and limited diffusion distance (a few hundred nanometers) of SO further confine the photodynamic reaction to regions in direct contact with the gel [58,59]. Consequently, the photodynamic effect remains localized at the gel–bacteria interface, resulting in a distinct and well-defined antibacterial zone. This strong retention confines the photodynamic response to the site of application, enabling precise and localized antibacterial action without systemic exposure. Such controlled, surface-limited activity makes the **RB**@Gel system particularly suitable for aPDT in skin and wound infections. A small clear area observed in the negative control was due to gel displacement rather than antibacterial activity.

The restricted photodynamic response of **RB**@Gel demonstrates selective antibacterial activity confined to the contact area, allowing targeted inactivation of *E. coli* while preserving the surrounding skin microbiota—an important feature for maintaining skin homeostasis and promoting wound healing [60,61]. Consequently, **RB**@Gel represents a promising supramolecular platform for targeted aPDT, suitable for treating localized infections such as contaminated wounds or postoperative lesions. Because the photodynamic mechanism relies on oxidative damage rather than specific molecular targets, this system could also be adapted for other bacterial species, including both Gram-negative and Gram-positive pathogens [62].

As a positive control, Kanamycin@Gel produced a 2.4 cm ZOI under both dark and light conditions, reflecting diffusion-based antibiotic activity. In contrast, the localized activity of **RB**@Gel ensures precise antibacterial action with minimal effect on surrounding tissues. Additionally, **RB** in solution showed no antibacterial activity without irradiation, but upon light exposure, 1 mM **RB** produced a 1.9 mm ZOI, confirming singlet oxygen generation and bacterial photoinactivation (Figure S3). The slightly larger ZOI in solution compared to the gel corresponds to higher diffusion, resulting in a broader but less localized effect. Overall, **RB**@Gel combines strong photosensitizer retention, sustained photodynamic efficacy, and excellent localization, making it an effective and biocompatible platform for topical antibacterial applications. Future work expanding the antibacterial assays will be essential to validate its full therapeutic potential across different infection models.

## 3. Materials and Methods

### 3.1. Materials

Rose Bengal, Kanamycin, absolute ethanol (≥99.9%), 9,10-anthracenedyl-bis(methylene)dimalonic acid (ABMA) and phosphate-buffered saline (PBS) tablets were purchased from Sigma-Aldrich (Schnelldorf, Germany). Ureadin Podos Gel Oil (Isdin Foot Care, Barcelona, Spain), Canesten cream (Lab Bayer S.A., Barcelona, Spain), Voltaren Emulgel (Novartis Pharmaceutics S.A., Barcelona, Spain) and SolvEasy Tinea Cream (Ego Pharmaceuticals, Braeside, Australia) were purchased. Milli-Q water (Millipore, Burlington, MA, USA) was obtained with Millipore’s Milli-Q Plus system and was used to prepare all samples. Nutrient Broth and Nutrient Agar were purchased from Condalab (Madrid, Spain). The synthesis of **1·2 Br** was carried out following a previously reported procedure [63].

### 3.2. AI Usage Statement

Illustrative schematics in Supplementary Information (Figures S4, S5, S7, S8 and S11) were generated using the Gemini 2.5 Pro model (Google, Mountain View, CA, USA).

### 3.3. Gels Preparation

The **1·2 Br**@Gel was prepared by mixing 500 µL of **1·2 Br** ethanolic solution with the concentration of 24 mM, followed by the addition of 500 µL of water to maintain an ethanol-to-water ratio of 1:1, yielding a final gel volume of 1 mL containing 12 mM **1·2 Br**. For the **RB**@Gel, a 4 mM stock solution of **RB** in ethanol was first prepared, and 250 µL of this solution were combined with 250 µL of the 48 mM **1·2 Br** ethanolic solution before adding 500 µL of water (ethanol-to-water ratio 1:1) to produce a final volume of 1 mL containing 1 mM **RB** and 12 mM **1·2 Br**. After gentle mixing, all samples were sealed and kept undisturbed at 25 °C. Gel formation was confirmed by the absence of flow when the vial was inverted.

### 3.4. Physicochemical Characterization

#### 3.4.1. Morphological Characterization

SEM images were obtained using a JEOL 7100F FEG-SEM (JEOL Ltd., Tokyo, Japan) at the *Centres Científics i Tecnològics de la Universitat de Barcelona* (CCiT-UB), Spain. A small portion of gel was placed on a borosilicate glass slide, mounted on an aluminum stub with carbon tape, and air-dried. Samples were coated with a 5 nm carbon layer prior to imaging at 5 kV.

#### 3.4.2. pH and Density

pH of the gels was measured with a pH meter (XS Vio Lab, Barcelona, Spain). 1 mL of the gel was dispersed in 9 mL of distilled water, followed by the treatment in an ultrasonic bath for 1 min to promote homogeneity [64]. Then, the electrode was inserted directly into it. Measurements were taken at room temperature (25 ± 2 °C). The results were expressed as mean ± standard deviation (n = 3).

The density (*ρ*) of the two gels was precisely determined using a standardized methodology based on the fundamental relationship *ρ* = *m*/*V*. Initially, a small cylinder of precisely known volume (*V*) was employed. Subsequently, the cylinder was filled with the gel sample, and the mass of the gel (*m*) was calculated by difference between the mass of the cylinder filled with gel and the mass of the empty cylinder. Finally, the calculated mass of each gel was divided by the cylinder’s volume to obtain the respective density values for the two gels under investigation. The results were expressed as mean ± standard deviation (n = 3).

#### 3.4.3. Swelling Test

The swelling ratio (*SR*) was determined gravimetrically. The pH values of 5.5 and 7.4 were selected for the hydrogel swelling characterization to represent physiologically relevant dermal microenvironments. The acidic condition of pH 5.5 was chosen to mimic the pH of intact, healthy skin (pH 4–6), reflecting the environment in which the gel would contact surrounding tissue under normal conditions. In contrast, the neutral condition of pH 7.4 simulates the interstitial fluid and acute wound exudate pH, which reflects the loss of the acidic barrier and serves as a baseline for the elevated alkaline conditions (pH 7.4–8.9) typically found in chronically infected or non-healing wounds [40]. Evaluating the gel’s swelling behavior at both pH 5.5 and pH 7.4 therefore enables assessment of its structural stability and therapeutic release profile during transition from healthy to a pathological dermal microenvironment, confirming its suitability for topical wound applications. Dried gels (30 mg) were placed in different Eppendorf tubes containing 1 mL of PBS (pH 5.5 and 7.4) and incubated at 32 °C for 20 min. After centrifugation (14,000 rpm, 10 min), the supernatant was removed and replaced with fresh PBS at different intervals for 10 min until equilibrium weight was achieved (Figure S4). *SR* was determined using the following equation:

(1) $$SR=\frac{Ws-W_{d}}{W_{d}},$$

where *Wₛ* and *W_d_* are the swollen and dry weights, respectively. The results were expressed as mean ± standard deviation (n = 3).

#### 3.4.4. Degradation Test

Degradation was assessed similarly to the swelling test but using freshly prepared gels (0.3 g). The percentage of weight loss (*WL*) was calculated using the following equation and analyzed through kinetic modeling:

(2) $$WL\;\left(\%\right)=\frac{W_{s}-W_{d}}{W_{d}}\times 100\;,$$

where *W_i_* is the initial gel weight and *W_d_* is the remaining weight at each interval. Data were expressed as mean ± standard deviation (n = 3).

#### 3.4.5. Extensibility Test

Extensibility of the gels were evaluated by placing 0.4–0.5 g of sample within a 1.76 cm^2^ area of the hole and applying sequential weights of 26, 36, 46, 76, 126, and 226 g for 2–3 min each at 25 °C (Figure S5). The test setup consisted of a metal extensometer plate with a grid surface, standard calibration weights, and a stainless-steel spatula for uniform gel spreading (Figure S6). The spread diameter was recorded and used to analyze the deformation behavior. Also, 0.44 mL of the different commercially available formulations were tested with a weight of 26 g in comparison with our gels. The results were expressed as mean ± standard deviation (n = 3).

#### 3.4.6. Rheological Measurements

Rheological tests were performed using a Haake RheoStress 1 rheometer (Thermo Fisher Scientific, Karlsruhe, Germany) with a P35 TiL plate geometry (35 mm diameter, 1 mm gap). Gels (with and without **RB**) were poured into 5.1 cm Petri dishes (3.6 mL) and stabilized for two days at room temperature. The samples were transferred carefully onto the lower plate, trimmed to match the plate surface, and analyzed at 25 °C. Oscillation amplitude tests were run at 1 Hz with shear stress increasing from 1 to 1000 Pa to determine the linear viscoelastic region (LVR).

#### 3.4.7. Release Studies

##### Dialysis Membrane Method

Pre-hydrated dialysis membranes (MWCO 10,000 Da, Thermo Scientific, Waltham, MA, USA) were used in a DMSO:H_2_O (1:9, *v*/*v*) medium under sink conditions. Gel samples were loaded into the membrane, sealed with parafilm, and immersed in the receptor phase. Samples were withdrawn at fixed intervals and replaced with equal volumes of fresh receptor medium (Figure S7). All tests were performed in triplicate; conditions are summarized in Table S8.

##### Direct-Surface Contact Method

**RB** release was further investigated using the direct-surface contact method as shown in Figure S8. The setup was mounted on a Microette system (Hanson Research, Chatsworth, CA, USA) connected to a thermostatic bath (32 ± 0.5 °C) [65]. Gels were directly immersed in DMSO:H_2_O (1:9) without a membrane barrier to evaluate unrestricted diffusion. Aliquots were collected at set times and replaced with fresh receptor medium. All experiments were triplicated, with conditions listed in Table S9.

##### Analytical Procedure for Release Studies

**RB** calibration curve (Figure S9) was obtained in DMSO:H_2_O (1:9) over the range 0.0099–20.35 µg/mL. Linear regression was applied to correlate absorbance and concentration. UV–Vis absorbance spectroscopy was used for quantification following validated protocol [66]. The kinetic data for **RB** release were analyzed by plotting the cumulative released amount against time. The resulting release profiles were then fitted to various kinetic models using GraphPad Prism software (version 5). The best-fit model was subsequently selected based on the highest Coefficient of Determination (R^2^) value.

#### 3.4.8. HET-CAM Irritancy Test

HET-CAM assay is a widely accepted in vitro technique for evaluating the irritation potential of substances, particularly those intended for ocular or topical use [67]. The method utilizes CAM of fertilized hen eggs, a highly vascularized and sensitive tissue, typically harvested after 9–10 days of incubation.

In this procedure, the eggs are carefully opened to expose the CAM, onto which the test and control gels are directly applied. A 0.9% sodium chloride (NaCl) solution is used as the negative control, as it does not induce irritation, while 0.1 N sodium hydroxide (NaOH) serves as the positive control, producing a rapid and severe reaction characterized by hemorrhage, vessel lysis, and coagulation. In the present study, **1·2 Br**@Gel and **RB**@Gel were assessed for their potential to cause irritation (Figure S10).

The appearance of vascular reactions such as hemorrhage, lysis, and coagulation was monitored and recorded at set time intervals (5, 30, and 300 s). The onset times of these events were then used to calculate an Irritation Score (IS) for each sample. Based on the IS values, the test materials were categorized into standard irritation levels (non-irritant, slightly irritant, moderately irritant, or severely irritant) (Table 2). This assay provides a reproducible and ethically responsible approach for determining the ocular safety of topical formulations [68].

The *IS* is calculated using this equation below:

(3) $$IS=\;\frac{(301\;-\;H)\;\times \;5\;+\;(301\;-\;L)\;\times \;7\;+\;(301\;-\;C)\;\times \;9}{300},$$

where *H*, *L*, and *C* denote the onset times (in seconds) of hemorrhage, lysis, and coagulation, respectively.

### 3.5. Ex Vivo Skin Permeation Studies

Ex vivo dorsal pig skin (Landrace × Large White, 40–45 kg) was obtained from surplus surgical animals following ethical approval (protocol 515/18, University of Barcelona). Animals were anesthetized (ketamine 3 mg/kg, xylazine 2.5 mg/kg, midazolam 0.17 mg/kg) and euthanized via sodium thiopental overdose. Skin samples were excised, immersed in PBS with 4% albumin and 10% DMSO, and stored at −80 °C. Before use, skin was thawed and sectioned to 700 µm thickness using a GA 630 dermatome (Aesculap, Tuttlingen, Germany), then mounted on Franz diffusion cells (Vidra Foc, Barcelona, Spain).

0.0506 g of **RB**@Gel was placed in the donor chamber, with the skin as a diffusion membrane and Milli-Q water as the receptor phase (Figure S11). After 24 h, the skin was removed, punctured, sonicated with 2 mL of Milli-Q water for 10 min, filtered (0.22 µm nylon), and analyzed. At the same time, receptor solution was also analyzed to quantify the **RB** that permeated through the skin. Experiments were performed in triplicate; conditions appear in Table S10.

#### Analytical Procedure for Ex Vivo Skin Permeation Studies

**RB** solutions were prepared in water, and the calibration curve (Figure S12) was made in the range of 0.19–12.5 µg/mL. The calibration curve was analyzed by linear regression method. Absorbance measurements were taken using Thermo Scientific™ Orion™ AquaMate 8100 spectrophotometer (Thermo Fisher Scientific, Waltham, MA, USA).

### 3.6. Optical Properties and SO Production

UV–Vis absorption spectra were recorded on a Thermo Scientific™ Orion™ AquaMate 8100 spectrophotometer (Thermo Fisher Scientific, Waltham, MA, USA), while fluorescence spectra were acquired using a Hitachi F-2710 spectrofluorimeter (Hitachi High-Tech, Tokyo, Japan). All spectroscopic measurements were carried out in quartz cuvettes (10 mm path length).

The sodium salt of ABMA (NaABMA) was prepared by dispersing ABMA in water and adding four mols of NaOH (from a stock solution with the concentration of 0.1 M) to ensure complete deprotonation. NaABMA acted as a chemical probe for singlet oxygen detection, with oxidation monitored as a decrease in its fluorescence emission upon irradiation.

Samples were irradiated in 10 mm quartz cuvettes for 30 min using a λ = 532 nm diode laser (50 mW, 5 V DC, 500 mA), corresponding to the absorption maximum of **RB**. Fluorescence spectra were acquired every 5 min from 400 to 550 nm, exciting at 380 nm. For each experiment, 0.1 mL of a 20 μM **RB** solution (prepared in a 1:1 ethanol–water mixture) or **RB**@Gel (same solvent composition) was mixed with 0.9 mL of 5 µM NaABMA in 2 mM PBS, giving a final **RB** concentration of 2 µM. All solutions were freshly prepared before use.

### 3.7. Antimicrobial PDT Efficacy

The antibacterial activity of **RB** in solution (1:1 water: ethanol) and in gel form **RB**@Gel was assessed against *E. coli* BL21 (DE3) using the Direct-Surface contact method. The *E. coli* BL21 (DE3) strain was used as a non-pathogenic laboratory model for proof-of-concept antibacterial testing due to its reproducible growth behavior and ease of handling. Although not a clinical isolate, it allows initial evaluation of the antimicrobial performance of the gel formulation. Sterile materials were used throughout. Treatments included: (i) **1·2 Br**@Gel (negative control), (ii) **RB**@Gel at 1000, 250, and 50 µM, and (iii) Kanamycin@Gel (positive control). Fresh bacterial cultures were grown in Luria broth at 37 °C and adjusted to OD_600_ = 0.5. A 200 µL aliquot of culture was spread evenly on nutrient agar plates, and 15 µL of gel sample was applied directly to the surface. For solution studies, sterile filter paper was immersed in **RB** solutions and placed on agar. Samples were irradiated at λ = 532 nm (50 mW power, operating voltage 5V DC, operating current 500 mA, Irradiance 10.2 kW/cm^2^, Fluence 18.36 MJ/cm^2^) for 30 min, then incubated at 37 °C for 24 h. The antibacterial effect was determined from the Zone of Inhibition (ZOI) measurements. The ZOI was measured as the width of the clear halo around the gel, excluding the diameter of the gel itself.

## 4. Conclusions

The supramolecular gels prepared in this work exhibit enhanced singlet oxygen generation under light irradiation, attributed to non-covalent interactions between Rose Bengal and the gelator within the supramolecular matrix. The resulting **RB**@Gel exhibited a nanofibrillar morphology, appropriate viscoelastic behavior, confirming their structural robustness and suitability for topical application. Spectroscopic and photochemical analyses demonstrated that **RB** retained its photophysical properties within the gel, while the supramolecular environment enhanced singlet oxygen generation efficiency compared with **RB** in solution. The degradation and swelling studies revealed distinct pH-responsive behavior, with faster erosion under acidic conditions due to electrostatic pairing between the negatively charged **RB** groups and the cationic imidazolium headgroups. At physiological pH, the gel network remained stable, ensuring persistence during topical application while enabling sustained disassembly over time. Such controlled degradation behavior supports prolonged contact with the infected area and facilitates a balance between structural integrity and gradual gel erosion, which is essential for topical aPDT performance.

Biological evaluation confirmed the relevance of **RB**@Gel for antimicrobial photodynamic therapy. The **RB**@Gel exhibited aPDT against *E. coli*, while maintaining negligible dark toxicity. The restricted diffusion of **RB**, together with its strong association within the supramolecular network, confines singlet oxygen generation to the gel–bacteria interface, minimizing damage to surrounding healthy tissue. This localized phototoxic effect is particularly beneficial for topical aPDT applications aimed at superficial infections. Furthermore, the HET-CAM test verified the non-irritant character of the gel, supporting its safety for cutaneous application. The combination of high photodynamic efficiency, biocompatibility, and structural tunability demonstrates the multifunctional nature of the designed **RB**@Gel system. Overall, the **RB**@Gel system provides several advantages, including localized retention of **RB** at the infection site, improved photodynamic efficiency, pH-dependent degradability, and excellent biocompatibility. Collectively, these findings highlight the potential of this supramolecular platform as a cost-effective and scalable approach for future topical photodynamic therapies targeting localized bacterial infections.

## Figures and Tables

**Table 1:** Storage modulus (G′), loss modulus (G″), and critical stress of gels obtained from stress sweep experiments at 25 °C. All measurements were performed at an oscillatory frequency of 1 Hz.

Sample	G′ (Pa) ^[a]^	G″ (Pa) ^[a]^	Critical Stress (Pa) ^[b]^
** 1·2 Br**@Gel	5923	1124	63
**RB**@Gel ^[c]^	58,394	13,395	423

^[a]^ Values correspond to the linear viscoelastic region, and ^[b]^ the critical stress represents the point at which G′ and G″ show a noticeable decrease. ^[c]^ **RB** concentration = 1 mM.

**Table 2:** Scoring system of HET-CAM test.

Irritation Score	Irritation Classification
0–0.9	Non-Irritant
1–4.9	Slight Irritant
5–8.9	Moderate Irritant
9–21	Severe Irritant

## Data Availability

The original contributions presented in this study are included in the article/Supplementary Material. Further inquiries can be directed to the corresponding author.

## Supplementary Materials

The following supporting information can be downloaded at https://www.mdpi.com/article/10.3390/ijms262311455/s1.
