# Supplementary material for: Rose Bengal-Incorporated Supramolecular Gels as a Topical Platform for Localized Antimicrobial Photodynamic Therapy
Source: Int J Mol Sci. 2025 Nov 26;26(23):11455. doi: 10.3390/ijms262311455 (PMC12692460; doi:10.3390/ijms262311455)
Supplement: Supplementary file 1 [file ijms-26-11455-s001.zip › ijms-3988418-supplementary.pdf]

SUPPLEMENTARY MATERIAL

Rose Bengal-incorporated supramolecular gels as a topical platform for localized antimicrobial photodynamic therapy

Kavya Anguluri[1,2], Saman Bagherpour[1,2], Ana C. Calpena[2,3], Lyda Halbaut[3], Alba Espargaró[3,4], Raimon Sabate[3,4], Lluïsa Pérez-García[1,2*]

[1] *Departament de Farmacologia, Toxicologia i Química Terapèutica, Facultat de Farmàcia i Ciències de l'Alimentació, Avda. Joan XXIII 27-31, Universitat de Barcelona, 08028 Barcelona, Spain*

[2] *Institut de Nanociència i Nanotecnologia IN2UB, Universitat de Barcelona, Barcelona, 08028 Spain*

[3] *Departament de Farmàcia, Tecnologia Farmacèutica, i Fisicoquímica, Facultat de Farmàcia i Ciències de l'Alimentació, Universitat de Barcelona, 08028 Barcelona, Spain*

[4] *Institut de Biomedicina (IBUB), Universitat de Barcelona, 08028 Barcelona, Spain*

Corresponding author's email: mlperez@ub.edu

INDEX

- Table S1: Statistical comparison of pH and density of **1·2 Br**@Gel and **RB**@Gel. Unpaired t-tests were performed to evaluate differences. Values include p-values, t-statistics, confidence intervals, and mean ± SEM for each group.
- Table S2: Swelling ratios of **RB**@Gel and **1·2 Br**@Gel at pH 5.5, presented as mean ± SD (N = 3). Statistical comparisons at each time point (1–9 min) were performed using unpaired t-test reporting p-values, t-statistics, confidence intervals, R^2 values, and mean ± SEM (N = 3) for each group.
- Table S3: Swelling ratios of **RB**@Gel and **1·2 Br**@Gel at pH 7.4, presented as mean ± SD (N = 3). Statistical comparisons at each time point (1–9 min) were performed using unpaired t-test reporting p-values, t-statistics, confidence intervals, R^2 values, and mean ± SEM (N = 3) for each group.
- Table S4: Parameters obtained from the one-site binding (hyperbolic) model describing the degradation kinetics of **1·2 Br**@Gel and **RB**@Gel at pH 5.5. Statistical comparison of their degradation behavior using unpaired t-tests. Reported values include p-values, t-statistics, confidence intervals, and mean ± SEM (N = 3) for each group.
- Table S5: Parameters obtained from the one-site binding (hyperbolic) model describing the degradation kinetics of **1·2 Br**@Gel and **RB**@Gel at pH 7.4. Statistical comparison of their degradation behavior using unpaired t-tests. Reported values include p-values, t-statistics, confidence intervals, and mean ± SEM (N = 3) for each group.
- Table S6A: Extensibility measurements of **1·2 Br**@Gel and **RB**@Gel under increasing applied weights, surface area values were presented as mean ± SD (N = 3). Statistical comparisons at each applied weight were performed using unpaired t-test reporting p-values, t-statistics, confidence intervals, and mean ± SEM (N = 3) for each group.
- Table S6B: Comparative and statistical analysis of extensibility values for **1·2 Br**@Gel, **RB**@Gel, and selected commercial formulations, using one-way ANOVA and Tukey's multiple comparison test. Surface area values were presented as mean ± SD (N = 3).
- Figure S1. Cumulative release profile of **RB** from gel using the direct-surface contact method.
- Table S7: Parameters obtained from the one-site binding (hyperbolic) model describing the release kinetics of **RB**@Gel using the direct-contact surface method.
- Figure S2: Emission spectra of ABMANa recorded upon irradiation.
- Figure S3: Representative agar plate images illustrating the aPDT activity of **RB** in solution under dark and light conditions.
- Figure S4: Procedure for performing the swelling test.
- Figure S5: Procedure for performing the extensibility test.
- Figure S6: Apparatus used for the extensibility test.
- Figure S7: Schematic representation of the release studies of the **RB** from gel using the dialysis membrane method setup.
- Table S8: Experimental conditions used for release studies in the membrane-assisted setup.
- Figure S8: Schematic representation of the release studies of the **RB** from gel using the direct-surface contact method setup.
- Table S9: Experimental conditions used for release studies in the direct-surface contact method.
- Figure S9: Calibration curve of **RB** in DMSO:H_2O (1:9) solution.
- Figure S10: Procedure for performing HET-CAM test on the gels.
- Figure S11: Schematic of the Franz diffusion cell setup used for the *ex-vivo* skin permeation study
- Table S10: Experimental conditions used for *ex-vivo* skin permeation studies.
- Figure S12: Calibration curve of **RB** in water.

Table S1: Statistical comparison of pH and density of **1·2 Br**@Gel and **RB**@Gel. Unpaired t-tests were performed to evaluate differences. Values include p-values, t-statistics, confidence intervals, and mean ± SEM for each group.

**Parameter**	**Value**	**Value**
Table Analyzed [a]	pH Columns A and B	Density Columns A and B
Unpaired t test		
P value	0.0056	0.0077
P value summary	**	**
Are means signif. different? ($P < 0.05$)	Yes	Yes
One- or two-tailed P value?	Two-tailed	Two-tailed
t, df	t=5.422, df=4	t=4.966, df=4
How big is the difference?		
Mean ± SEM of column A	5.200 ± 0.05774 N=3	1.159 ± 0.03060 N=3
Mean ± SEM of column B	5.900 ± 0.1155 N=3	0.9540 ± 0.02771 N=3
Difference between means	-0.7000 ± 0.1291	0.2050 ± 0.04128
95% confidence interval	0.3416 to 1.058	-0.3196 to -0.09040
R squared	0.8802	0.8604
F test to compare variances		
F, DFn, Dfd	4.000, 2, 2	1.219, 2, 2
P value	0.2	0.4506
P value summary	ns	ns
Are variances significantly different?	No	No

[a] Column A: **RB**@Gel, Column B: **1·2 Br**@Gel.

Table S2: Swelling ratios of **RB**@Gel and **1:2 Br**@Gel at different time points (pH 5.5), presented as mean ± SD (N = 3). Unpaired t-test p-values for comparisons between the two groups at each time point were also provided.

Time (min)	RB@Gel	1·2 Br@Gel	p-value
1	3.30 ± 0.37	4.70 ± 0.50	0.0176
2	5.79 ± 0.65	5.06 ± 0.54	0.2111
3	5.57 ± 0.63	6.01 ± 0.64	0.4463
4	3.26 ± 0.37	2.13 ± 0.23	0.0104
6	2.80 ± 0.32	2.15 ± 0.23	0.0442
9	2.28 ± 0.26	3.33 ± 0.36	0.0142

Statistical comparison of swelling ratios (at 1, 2, 3, 4, 6, and 9 min) between Columns A and B. Unpaired t-tests and F-tests were performed to evaluate differences. Values include p-values, t-statistics, confidence intervals, R^2 values, and mean ± SEM (N = 3) for each group.

[a] Column A: **RB**@Gel, Column B: **1·2 Br**@Gel.

**Parameter**	**Value**	**Value**	**Value**	**Value**	**Value**	**Value**
**Table Analyzed**[a]	**Swelling 1 Columns A and B**	**Swelling 2 Columns A and B**	**Swelling 3 Columns A and B**	**Swelling 4 Columns A and B**	**Swelling 6 Columns A and B**	**Swelling 9 Columns A and B**
Unpaired t test						
P value	0.0176	0.2111	0.4463	0.0104	0.0442	0.0142
P value summary	*	ns	ns	*	*	*
Are means signif. different? (P < 0.05)	Yes	No	No	Yes	Yes	Yes
One- or two-tailed P value?	Two-tailed	Two-tailed	Two-tailed	Two-tailed	Two-tailed	Two-tailed
t, df	t=3.897 df=4	t=1.487 df=4	t=0.8438 df=4	t=4.555 df=4	t=2.897 df=4	t=4.155 df=4
How big is the difference?						
Mean ± SEM of column A	3.295 ± 0.2140 N=3	5.792 ± 0.3762 N=3	5.575 ± 0.3621 N=3	3.264 ± 0.2120 N=3	2.801 ± 0.1819 N=3	2.276 ± 0.1479 N=3
Mean ± SEM of column B	4.703 ± 0.2912 N=3	5.064 ± 0.3135 N=3	6.013 ± 0.3722 N=3	2.127 ± 0.1317 N=3	2.148 ± 0.1330 N=3	3.331 ± 0.2062 N=3
Difference between means	-1.408 ± 0.3614	0.7284 ± 0.4897	-0.4382 ± 0.5193	1.137 ± 0.2496	0.6529 ± 0.2254	-1.054 ± 0.2537
95% confidence interval	0.4052 to 2.412	-2.088 to 0.6310	-1.003 to 1.880	-1.830 to -0.4440	-1.279 to -0.02730	0.3498 to 1.758
R squared	0.7915	0.3561	0.1511	0.8384	0.6772	0.8119
F test to compare variances						
F,DFn, Dfd	1.851, 2, 2	1.440, 2, 2	1.057, 2, 2	2.592, 2, 2	1.871, 2, 2	1.945, 2, 2
P value	0.3507	0.4098	0.4862	0.2784	0.3483	0.3396
P value summary	ns	ns	ns	ns	ns	ns
Are variances significantly different?	No	No	No	No	No	No

Table S3: Swelling ratios of **RB**@Gel and **1:2 Br**@Gel at different time points (pH 7.4), presented as mean ± SD (N = 3). Unpaired t-test p-values for comparisons between the two groups at each time point were also provided.

Time (min)	RB@Gel	1·2 Br@Gel	p-value
1	3.15 ± 0.35	2.52 ± 0.27	0.0698
2	3.46 ± 0.38	5.09 ± 0.54	0.0136
3	3.37 ± 0.41	4.87 ± 0.52	0.0334
4	4.39 ± 0.49	4.88 ± 0.52	0.3046
6	4.32 ± 0.48	4.88 ± 0.52	0.2494
9	2.63 ± 0.29	5.10 ± 0.54	0.0023

Statistical comparison of swelling ratios (at 1, 2, 3, 4, 6, and 9 min) between Columns A and B. Unpaired t-test was performed to evaluate differences. Values include p-values, t-statistics, confidence intervals, R^2 values, and mean ± SEM (N = 3) for each group.

**Parameter**	**Value**	**Value**	**Value**	**Value**	**Value**	**Value**
**Table Analyzed[a]**	**Swelling 1 Columns A and B**	**Swelling 2 Columns A and B**	**Swelling 3 Columns A and B**	**Swelling 4 Columns A and B**	**Swelling 6 Columns A and B**	**Swelling 9 Columns A and B**
Unpaired t test						
P value	0.0698	0.0136	0.0334	0.3046	0.2494	0.0023
P value summary	ns	*	*	ns	ns	**
Are means signif. different? (P < 0.05)	No	Yes	Yes	No	No	Yes
One- or two-tailed P value?	Two-tailed	Two-tailed	Two-tailed	Two-tailed	Two-tailed	Two-tailed
t, df	t=2.459 df=4	t=4.212 df=4	t=3.186 df=4	t=1.177 df=4	t=1.347 df=4	t=6.874 df=4
How big is the difference?						
Mean ± SEM of column A	3.158 ± 0.2051 N=3	3.464 ± 0.2250 N=3	3.650 ± 0.2371 N=3	4.391 ± 0.2852 N=3	4.328 ± 0.2811 N=3	2.634 ± 0.1711 N=3
Mean ± SEM of column B	2.524 ± 0.1563 N=3	5.096 ± 0.3155 N=3	4.872 ± 0.3016 N=3	4.880 ± 0.3021 N=3	4.884 ± 0.3024 N=3	5.104 ± 0.3160 N=3
Difference between means	0.6340 ± 0.2579	-1.632 ± 0.3875	-1.222 ± 0.3836	-0.4888 ± 0.4155	-0.5559 ± 0.4129	-2.470 ± 0.3593
95% confidence interval	-1.350 to 0.08181	0.5566 to 2.708	0.1572 to 2.287	-0.6645 to 1.642	-0.5902 to 1.702	1.472 to 3.467
R squared	0.6018	0.816	0.7173	0.2571	0.3119	0.9219
F test to compare variances						
F,DFn, Dfd	1.723, 2, 2	1.967, 2, 2	1.619, 2, 2	1.122, 2, 2	1.157, 2, 2	3.411, 2, 2
P value	0.3672	0.3371	0.3819	0.4712	0.4636	0.2267
P value summary	ns	ns	ns	ns	ns	ns
Are variances significantly different?	No	No	No	No	No	No

[a] Column A: **RB**@Gel, Column B: **1·2 Br**@Gel.

Table S4: Parameters obtained from the one-site binding (hyperbolic) model describing the degradation kinetics of **1·2 Br**@Gel and **RB**@Gel at pH 5.5.

	**RB@Gel**	**1·2 Br@Gel**
One site binding (hyperbola)	$Y = Bmax_1 \cdot X/(Kd_1 + X)$	
Best-fit values		
**Bmax**	79.84	33.26
**Kd**	65.08	103.5
Std. Error		
**Bmax**	6.014	2.130
**Kd**	13.57	15.36
95% Confidence Intervals		
**Bmax**	67.09 to 92.59	28.75 to 37.78
**Kd**	36.31 to 93.85	70.92 to 136.1
Goodness of Fit		
Degrees of Freedom	16	16
R^2	0.9439	0.9716

Statistical comparison of degradation behavior of **1·2 Br**@Gel and **RB**@Gel at pH 5.5. Unpaired t-test was performed to evaluate differences. Values include p-values, t-statistics, confidence intervals, and mean ± SEM (N = 3) for each group.

**Parameter**[a]	**Value**	**Value**
Table Analyzed	**Bmax** Columns A and B	**Kd** Columns A and B
Unpaired t test		
P value	0.0019	0.1341
P value summary	**	ns
Are means signif. different? (P < 0.05)	Yes	No
One- or two-tailed P value?	Two-tailed	Two-tailed
t, df	t=7.305 df=4	t=1.875 df=4
How big is the difference?		
Mean ± SEM of column A	79.84 ± 6.010 N=3	65.08 ± 13.57 N=3
Mean ± SEM of column B	33.26 ± 2.130 N=3	103.5 ± 15.36 N=3
Difference between means	46.58 ± 6.377	-38.42 ± 20.49
95% confidence interval	-64.28 to -28.88	-18.47 to 95.31
R squared	0.9303	0.4677
F test to compare variances		
F,DFn, Dfd	7.959, 2, 2	1.281, 2, 2
P value	0.1116	0.4384
P value summary	ns	ns
Are variances significantly different?	No	No

[a] Column A: **RB**@Gel, Column B: **1·2 Br**@Gel.

Table S5: Parameters obtained from the one-site binding (hyperbolic) model describing the degradation kinetics of **1·2 Br**@Gel and **RB**@Gel at pH 7.4.

	**RB@Gel**	**1.2 Br@Gel**
One site binding (hyperbola)	$Y = Bmax_1 \cdot X/(Kd_1 + X)$	
Best-fit values		
**Bmax**	59.36	50.64
**Kd**	37.95	25.13
Std. Error		
**Bmax**	3.949	3.16
**Kd**	10.04	7.514
95% Confidence Intervals		
**Bmax**	49.21 to 69.51	42.52 to 58.77
**Kd**	12.14 to 63.76	5.807 to 44.44
Goodness of Fit		
Degrees of Freedom	5	5
R^2	0.9883	0.9871
Absolute Sum of Squares	25.87	22.12
Sy.x	2.275	2.103
Data		
Number of X values	7	7
Number of Y replicates	1	1
Total number of values	7	7
Number of missing values	0	0

Statistical comparison of degradation behavior of **1·2 Br**@Gel and **RB**@Gel at pH 5.5. Unpaired t-test was performed to evaluate differences. Values include p-values, t-statistics, confidence intervals, and mean ± SEM (N = 3) for each group.

**Parameter**	**Value**	**Value**
Table analyzed[a]	**Bmax** Columns A and B	**Kd** Columns A and B
Unpaired t test		
P value	0.1594	0.3642
P value summary	ns	ns
Are means signif. different? (P < 0.05)	No	No
One- or two-tailed P value?	Two-tailed	Two-tailed
t, df	t=1.726 df=4	t=1.023 df=4
How big is the difference?		
Mean ± SEM of column A	59.36 ± 3.943 N=3	37.95 ± 10.03 N=3
Mean ± SEM of column B	50.64 ± 3.158 N=3	25.13 ± 7.511 N=3
Difference between means	8.720 ± 5.052	12.82 ± 12.53
95% confidence interval	-22.74 to 5.305	-47.62 to 21.98
R squared	0.4269	0.2073
F test to compare variances		
F,DFn, Dfd	1.559, 2, 2	1.785, 2, 2
P value	0.3908	0.3591
P value summary	ns	ns
Are variances significantly different?	No	No

[a] Column A: **RB**@Gel, Column B: **1·2 Br**@Gel.

Table S6A: Extensibility measurements of **1·2 Br**@Gel and **RB**@Gel under increasing applied weights. Surface area values and unpaired t-test p-values were shown for each applied load. The values were expressed as mean ± SD (N = 3).

	**1·2 Br@Gel**	**RB@Gel**	**Unpaired t test**
**Weight (g)**	**Surface area (cm^2)**		**P value**
0	2.835287 ± 0.5	2.835287 ± 0.5	-
26.06	4.90625 ± 0.49	5.72555 ± 0.48	0,1074
36.06	4.90625 ± 0.5	5.72555 ± 0.57	0,1346
46.06	5.3066 ± 0.52	5.72555 ± 0.53	0,3838
76.06	5.72265 ± 0.56	5.72555 ± 0.55	0,9952
126.06	5.72265 ± 0.57	5.72555 ± 0.58	0,9954
226.06	6.1544 ± 0.61	5.72555 ± 0.62	0,4412

Parameters obtained from unpaired t-test comparing the release profiles of **1·2 Br**@Gel and **RB**@Gel at different applied weights (26.06–226.06 g). Unpaired t-test was performed to evaluate differences. Values include p-values, t-statistics, confidence intervals, and mean ± SEM (N = 3) for each group.

**Table Analyzed**[a]	**26.06 g**	**36.06 g**	**46.06 g**	**76.06 g**	**126.06 g**	**226.06 g**
Column A	(**1·2 Br**@Gel)	(**1·2 Br**@Gel)	(**1·2 Br**@Gel)	(**1·2 Br**@Gel)	(**1·2 Br**@Gel)	(**1·2 Br**@Gel)
vs	vs	vs	vs	vs	vs	vs
Column B	**RB**@Gel	**RB**@Gel	**RB**@Gel	**RB**@Gel	**RB**@Gel	**RB**@Gel
Unpaired t test						
**P value**	**0,1074**	**0,1346**	**0,3838**	**0,9952**	**0,9954**	**0,4412**
P value summary	ns	ns	ns	ns	ns	ns
Are means signif. different? (P < 0.05)	No	No	No	No	No	No
One- or two-tailed P value?	Two-tailed	Two-tailed	Two-tailed	Two-tailed	Two-tailed	Two-tailed
t, df	t=2.069 df=4	t=1.872 df=4	t=0.9773 df=4	t=0.006406 df=4	t=0.006183 df=4	t=0.8540 df=4
How big is the difference?						
Mean ± SEM of column A	4.906 ± 0.2829 N=3	4.906 ± 0.2887 N=3	5.307 ± 0.3002 N=3	5.723 ± 0.3233 N=3	5.723 ± 0.3291 N=3	6.154 ± 0.3522 N=3
Mean ± SEM of column B	5.726 ± 0.2771 N=3	5.726 ± 0.3291 N=3	5.726 ± 0.3060 N=3	5.726 ± 0.3175 N=3	5.726 ± 0.3349 N=3	5.726 ± 0.3580 N=3
Difference between means	-0.8193 ± 0.3960	-0.8193 ± 0.4378	-0.4190 ± 0.4287	-0.002903 ± 0.4532	-0.002903 ± 0.4695	0.4288 ± 0.5022
95% confidence interval	-1.919 to 0.2801	-2.035 to 0.3959	-1.609 to 0.7711	-1.261 to 1.255	-1.306 to 1.300	-0.9652 to 1.823
R squared	0,5169	0,4669	0,1928	0,00001026	0,000009558	0,1542
F test to compare variances						
F,DFn, Dfd	1.042, 2, 2	1.300, 2, 2	1.039, 2, 2	1.037, 2, 2	1.035, 2, 2	1.033, 2, 2
P value	0,9794	0,8697	0,9810	0,9820	0,9826	0,9837
P value summary	ns	ns	ns	ns	ns	ns
Are variances significantly different?	No	No	No	No	No	No

[a] Column A: **1·2 Br** @Gel, Column B: **RB**@Gel.

Table S6B: Comparative extensibility values of both the **1·2 Br**@Gel and **RB**@Gel with selected commercial topical formulations. The values were expressed as mean $\pm$ SD (N=3).

Sample	Surface Area (cm^2)
**1·2 Br**@Gel	4,91 ± 0,49
**RB**@Gel	5,72± 0,48
Ureadin podos	15,89 ± 1,36
Canesten	7,06 ± 0,84
Voltaren	12,56 ± 1,53
SolvEasy Tinea	7,06 ± 0,76

Statistical comparison of extensibility values of **1·2 Br**@Gel and **RB**@Gel with selected commercial topical formulations. One-way ANOVA was performed to determine overall differences among the six formulations, followed by Tukey's multiple comparison test to identify significant pairwise differences. Values include mean differences, q-statistics, significance levels, and confidence intervals for each comparison.

**ANOVA Table**	**SS**	**df**	**MS**		
Treatment (between columns)	285,2	5	57,03		
Residual (within columns)	11,89	12	0,9907		
Total	297,0	17			
Tukey's Multiple Comparison Test	Mean Diff.	q	Significant? P < 0.05?	Summary	95% CI of diff
1-2 Br@Gel vs RB@Gel	-0,8100	1,410	No	ns	-3.540 to 1.920
1-2 Br@Gel vs Ureadin podos	-10,98	19,11	Yes	***	-13.71 to -8.250
1-2 Br@Gel vs Canesten	-2,150	3,741	No	ns	-4.880 to 0.5802
1-2 Br@Gel vs Voltaren	-7,650	13,31	Yes	***	-10.38 to -4.920
1-2 Br@Gel vs SolvEasy Tinea	-2,150	3,741	No	ns	-4.880 to 0.5802
RB@Gel vs Ureadin podos	-10,17	17,70	Yes	***	-12.90 to -7.440
RB@Gel vs Canesten	-1,340	2,332	No	ns	-4.070 to 1.390
RB@Gel vs Voltaren	-6,840	11,90	Yes	***	-9.570 to -4.110
RB@Gel vs SolvEasy Tinea	-1,340	2,332	No	ns	-4.070 to 1.390
Ureadin podos vs Canesten	8,830	15,37	Yes	***	6.100 to 11.56
Ureadin podos vs Voltaren	3,330	5,795	Yes	*	0.5998 to 6.060
Ureadin podos vs SolvEasy Tinea	8,830	15,37	Yes	***	6.100 to 11.56
Canesten vs Voltaren	-5,500	9,571	Yes	***	-8.230 to -2.770
Canesten vs SolvEasy Tinea	0,0000	0,0000	No	ns	-2.730 to 2.730
Voltaren vs SolvEasy Tinea	5,500	9,571	Yes	***	2.770 to 8.230

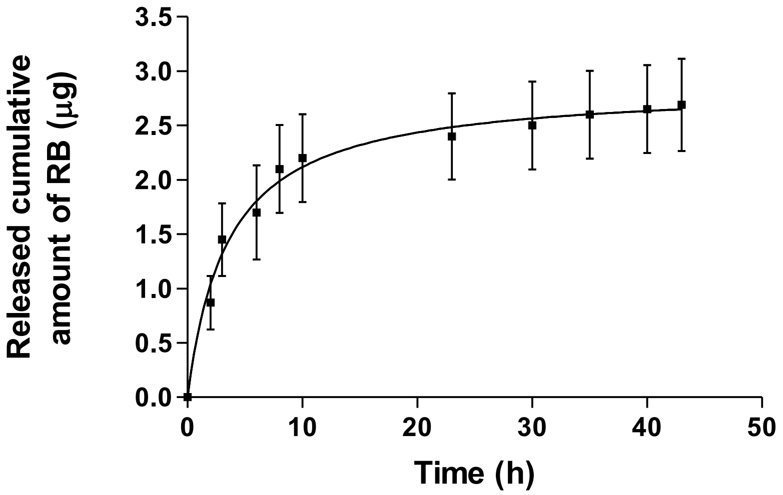

Figure S1. Cumulative release profile of **RB** from the gel using the direct-surface contact method. Data are expressed as mean ± SD (n = 3).

Table S7: Parameters obtained from the one-site binding (hyperbolic) model describing the release kinetics of **RB** from gel using the direct-contact surface method.

	**RB@Gel**
One site binding (hyperbola) $Y = Bmax_1 \cdot X/(Kd_1 + X)$	
Best-fit values	
**Bmax**	2.866
**Kd**	3.54
Std. Error	
**Bmax**	0.06678
**Kd**	0.3689
95% Confidence Intervals	
**Bmax**	2.715 to 3.017
**Kd**	2.705 to 4.374
Goodness of Fit	
Degrees of Freedom	9
R^2	0.996

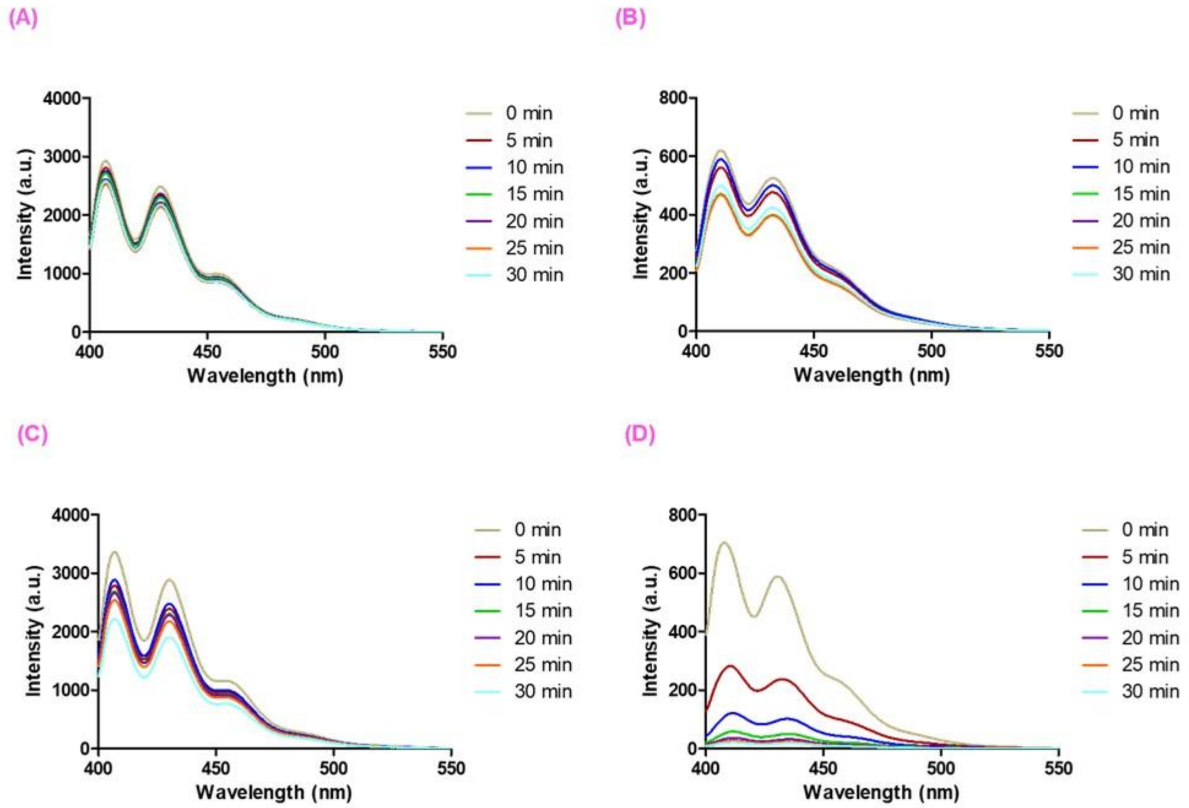

Figure S2: Emission spectra of ABMANa recorded upon irradiation. NaABMA – In Solution (A) & In **1·2 Br**@Gel (B), **RB** – In Solution (C) & In Gel (D).

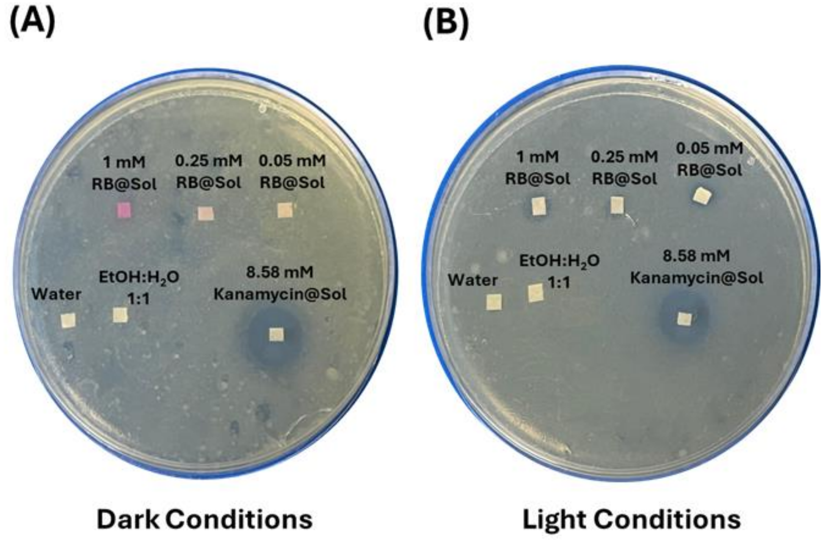

Figure S3: Representative agar plate images illustrating the aPDT activity of **RB** in solution under dark and light conditions. The concentration of the gelator is 12 mM in all the gels.

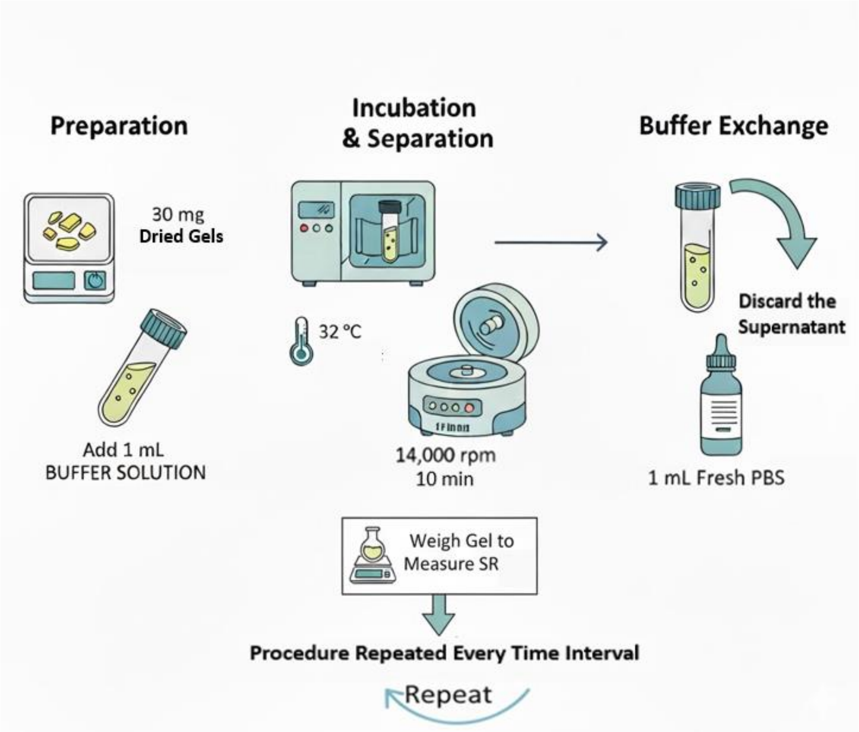

Figure S4: Procedure for performing the swelling test.

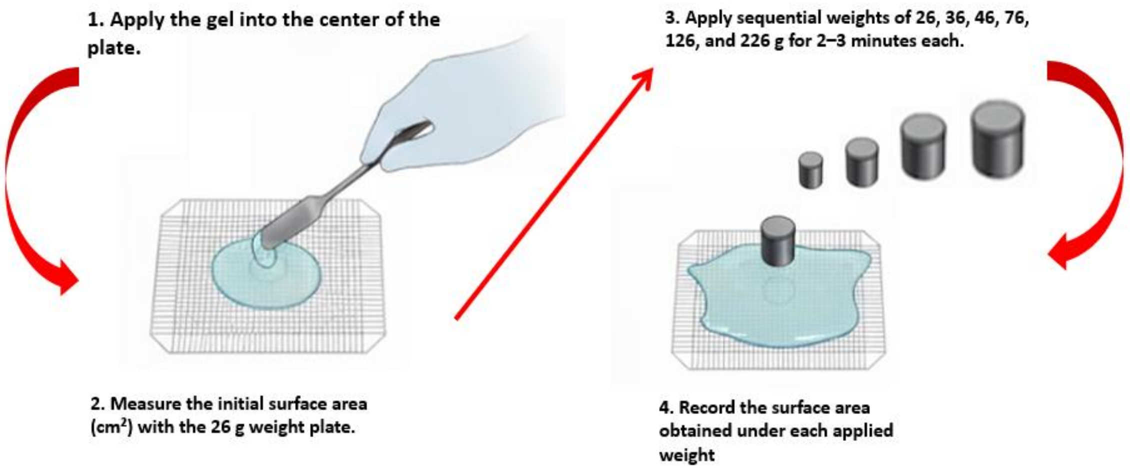

Figure S5: Procedure for performing the extensibility test.

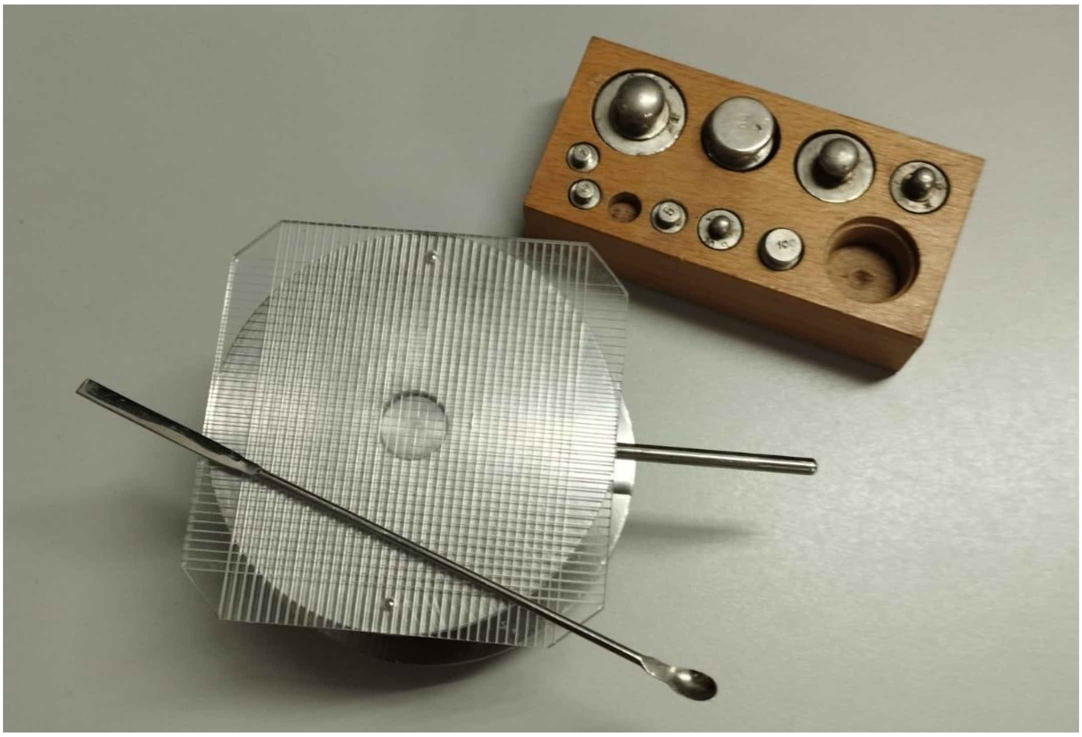

Figure S6: Apparatus used for the extensibility test, consisting of a metal extensometer plate with a grid surface, standard calibration weights, and a stainless-steel spatula for uniform gel spreading.

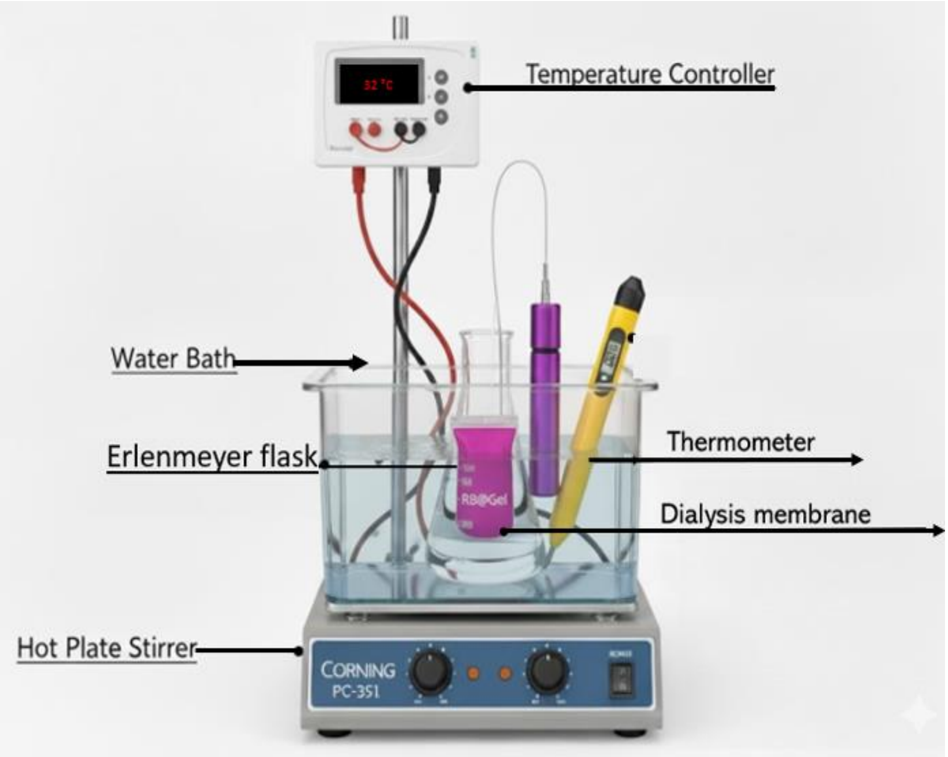

Figure S7: Schematic representation of the release studies of the **RB** from gel using the dialysis membrane method setup.

Table S8: Experimental conditions used for release studies in the membrane-assisted setup.

**Parameters**	**Description**
Cell volume	500 mL
Diffusion area	15. 08 cm^2
Time points	0, 2, 4, 6, 8, 24, 26, 28, 30, 32, 48, 50, 52, 54, 56, 72, 74, 76, 78, 80, 144 and 146 hrs
Replicates	3
Membrane	Thermoscientific dialysis membrane, MWCO (10,000 Da)
Stirring	550 rpm
Receptor fluid	DMSO: H2O (1:9)
Temperature	32 $^oC \pm 0.5\ ^oC$
Receptor Phase Sampling volume	1000 μL
Dose	**RB** – 3.34 mL (305.292 μg/mL)

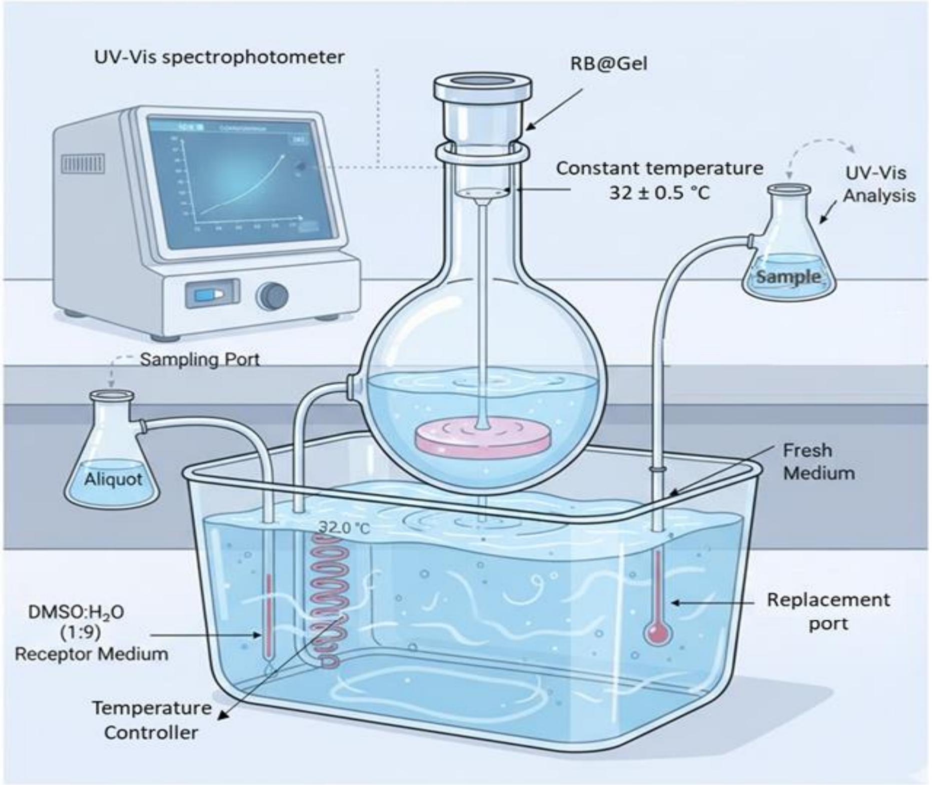

Figure S8: Schematic representation of the release studies of the **RB** from gel using the direct-surface contact method setup.

Table S9: Experimental conditions used for release studies in the direct-surface contact method.

Conditions	Description
Round Bottom flask volume	10 mL
Time intervals	0, 2, 3, 6, 8, 10, 23, 30, 35, 40, 43 hrs
Replicates	3
Stirring	180 rpm
Receptor fluid	DMSO: H2O (1:9)
Temperature	32 $^{o}C \pm 0.5$ ^{o}C
Sampling volume	1 mL
Dose	**RB** – 0.203 mL (206.57 μg/mL)

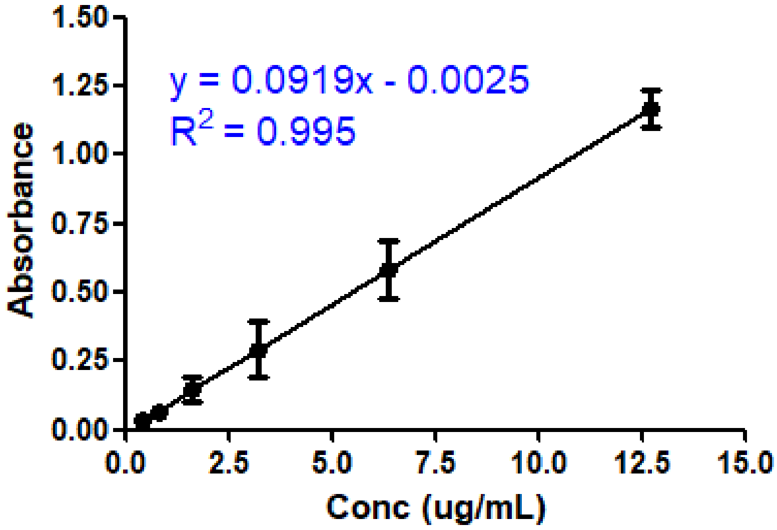

Figure S9: Calibration curve of **RB** in DMSO:H_2O (1:9) solution.

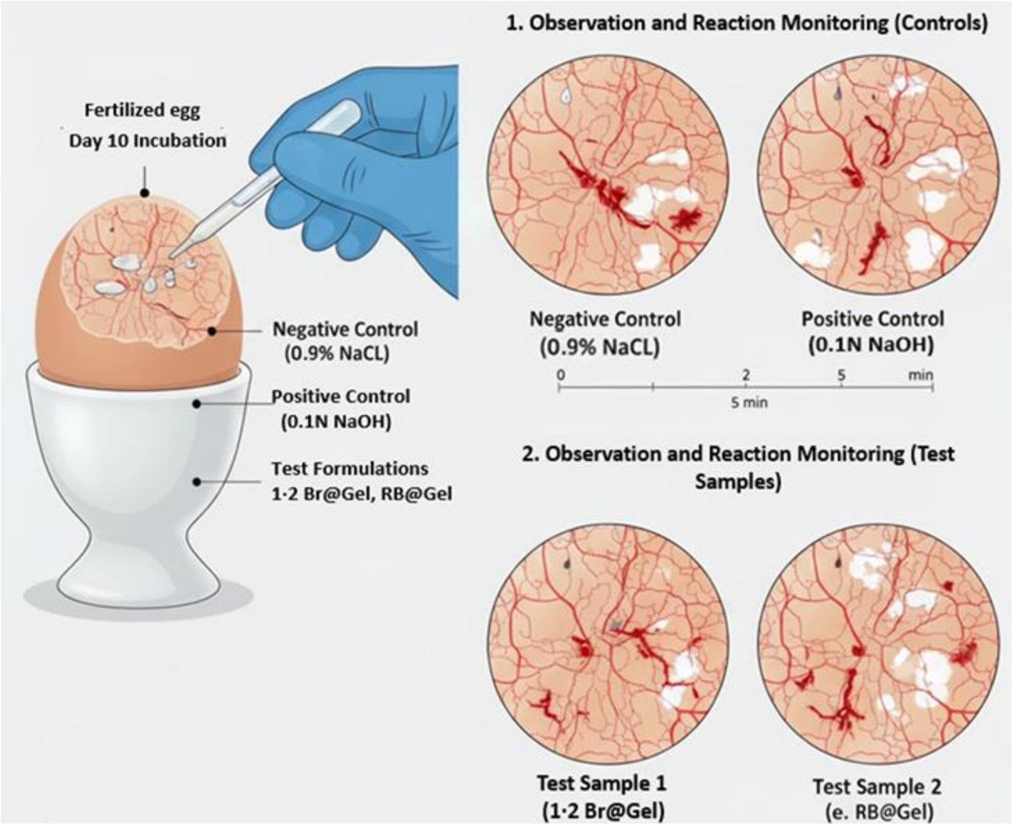

Figure S10: Procedure for performing HET-CAM test on the gels.

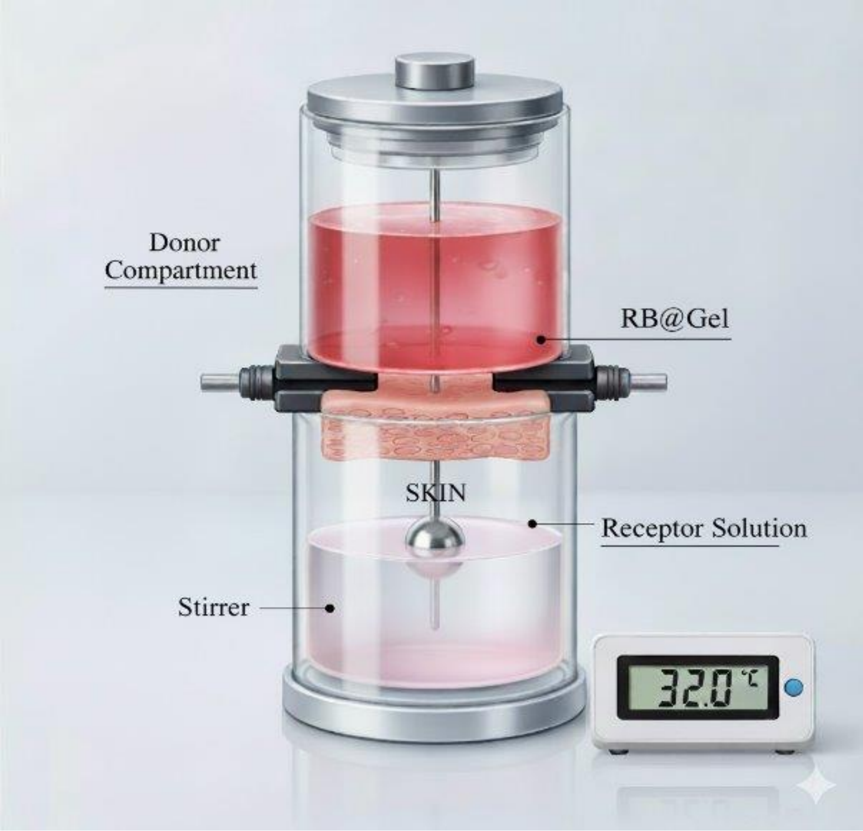

Figure S11: Schematic representation of the Franz diffusion cell setup used for the *ex-vivo* skin permeation study.

Table S10: Experimental conditions used for *ex-vivo* skin permeation studies.

Parameters	Description
Skin type	dorsal pig skin
Replicates	3
Diffusion area	1.1879 cm^2
**RB**@Gel in donor phase	0.0506 g (50.6 mg)
Receptor volume	5 mL water
Density of gel	1.159 g/mL
Applied gel volume	0.0437 mL
Dose normalized	0.33 μg/cm^2
Receptor Temperature	32 $^oC \pm 0.5$ ^oC
Exposure duration	24 h

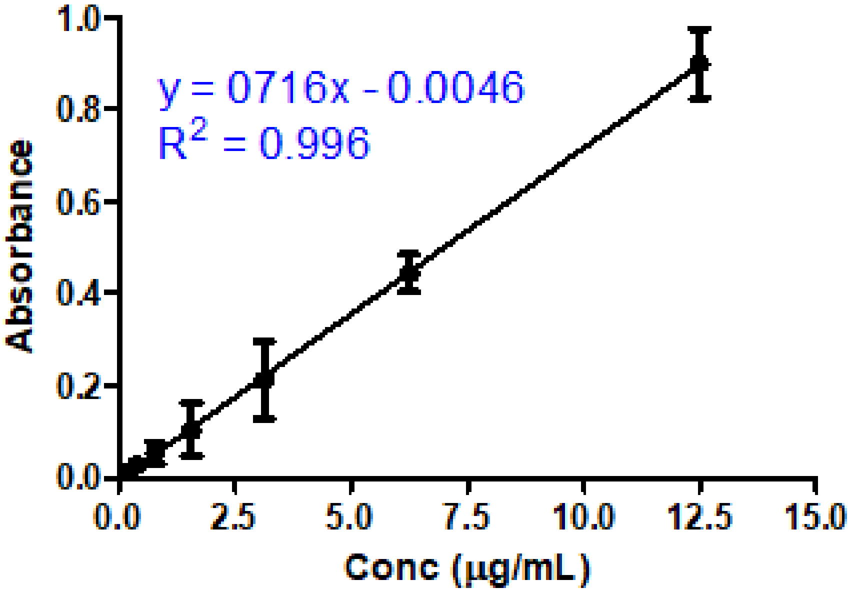

Figure S12: Calibration curve of **RB** in water.
